# Haplotype Shuffling and Dimorphic Transposable Elements in the Human Extended Major Histocompatibility Complex Class II Region

**DOI:** 10.3389/fgene.2021.665899

**Published:** 2021-05-28

**Authors:** Jerzy K. Kulski, Shingo Suzuki, Takashi Shiina

**Affiliations:** ^1^Faculty of Health and Medical Sciences, The University of Western Australia, Crawley, WA, Australia; ^2^Department of Molecular Life Sciences, Division of Basic Medical Science and Molecular Medicine, Tokai University School of Medicine, Isehara, Japan

**Keywords:** major histocompatibility complex, haplotypes, DNA sequences, Retroelements, single-nucleotide polymorphism-density crossovers, polymorphisms, indels, shuffling

## Abstract

The major histocompatibility complex (MHC) on chromosome 6p21 is one of the most single-nucleotide polymorphism (SNP)-dense regions of the human genome and a prime model for the study and understanding of conserved sequence polymorphisms and structural diversity of ancestral haplotypes/conserved extended haplotypes. This study aimed to follow up on a previous analysis of the MHC class I region by using the same set of 95 MHC haplotype sequences downloaded from a publicly available BioProject database at the National Center for Biotechnology Information to identify and characterize the polymorphic *human leukocyte antigen (HLA)-*class II genes, the *MTCO3P1* pseudogene alleles, the indels of transposable elements as haplotypic lineage markers, and SNP-density crossover (XO) loci at haplotype junctions in DNA sequence alignments of different haplotypes across the extended class II region (∼1 Mb) from the telomeric *PRRT1* gene in class III to the *COL11A2* gene at the centromeric end of class II. We identified 42 haplotypic indels (20 Alu, 7 SVA, 13 LTR or MERs, and 2 indels composed of a mosaic of different transposable elements) linked to particular HLA-class II alleles. Comparative sequence analyses of 136 haplotype pairs revealed 98 unique XO sites between SNP-poor and SNP-rich genomic segments with considerable haplotype shuffling located in the proximity of putative recombination hotspots. The majority of XO sites occurred across various regions including in the vicinity of *MTCO3P1* between *HLA-DQB1* and *HLA-DQB3*, between *HLA-DQB2* and *HLA-DOB*, between *DOB* and *TAP2*, and between *HLA-DOA* and *HLA-DPA1*, where most XOs were within a *HERVK22* sequence. We also determined the genomic positions of the PRDM9-recombination suppression sequence motif *ATCCATG/CATGGAT* and the PRDM9 recombination activation partial binding motif *CCTCCCCT/AGGGGAG* in the class II region of the human reference genome (NC_ 000006) relative to published meiotic recombination positions. Both the recombination and anti-recombination PRDM9 binding motifs were widely distributed throughout the class II genomic regions with 50% or more found within repeat elements; the anti-recombination motifs were found mostly in L1 fragmented repeats. This study shows substantial haplotype shuffling between different polymorphic blocks and confirms the presence of numerous putative ancestral recombination sites across the class II region between various HLA class II genes.

## Introduction

Haplotypes are combinations of alleles at different loci of phased DNA segregating together in multigenerational families (Bodmer et al., 1986; Lloyd et al., 2016; Alper and Larsen, 2017) essentially as DNA sequences that are identical by descent (IBD) *via* recent shared ancestry (Druet and Farnir, 2011; Browning and Browning, 2012; Thompson, 2013; Zhou et al., 2020b). The word haplotype (single, from haploid) was first introduced by Ruggero Ceppellini in 1966/67 to describe immunoglobin allotypes as corresponding “to the product of a single gene dose” and was appropriated almost immediately by immunogeneticists to describe the linked alleles in the highly polymorphic, multilocus human major histocompatibility complex (MHC) super locus on chromosome 6 (Bodmer, 2019) that consists of three distinct genomic regions, classes I, II and III with clusters of human leukocyte antigen (HLA) genes involved in the regulation of the innate and adaptive immune system, autoimmunity, and transplantation (Shiina et al., 2004, 2009; Vandiedonck and Knight, 2009; Trowsdale, 2011). During the past 30 years, the study of human MHC population haplotypes for transplantation and disease has developed into a formidable field of segregated haplotype blocks analyzed by congruence (Baschal et al., 2012) and conserved polymorphic sequences (CPSs) of ancestral haplotypes (AH), and conserved extended haplotypes (CEHs) (Awdeh et al., 1983; Dawkins et al., 1983, 1999; Contu et al., 1989; Degli-Esposti et al., 1992; Yunis et al., 2003; Alper et al., 2006; Aly et al., 2006; Bilbao et al., 2006; Smith et al., 2006; Lam et al., 2013; Gambino et al., 2018). After the transition into the third millennium and the publication of the analysis of the first human genomic sequence (International Human Genome Sequencing Consortium, 2001; Venter et al., 2001), haplotype studies began to spread in earnest from the continuous analysis of the MHC super locus (Jeffreys et al., 2001; Ahmad et al., 2003; Kauppi et al., 2003; Miretti et al., 2005; Blomhoff et al., 2006) to other regions of the human genome (Daly et al., 2001; Gabriel et al., 2002; Jeffreys et al., 2004; Kauppi et al., 2004; Conrad et al., 2006; The International HapMap Consortium, 2007; Baschal et al., 2012; Browning and Browning, 2020; Nait Saada et al., 2020) and across to other species (Guryev et al., 2006; Kauppi et al., 2007; Villa-Angulo et al., 2009; Ando et al., 2019; Lan et al., 2019). Genomic haplotype blocks are now more commonly described in terms of haplotype estimations using the less structurally precise population linkage disequilibrium (LD) statistics and inferred LD-allelic block analyses (Al Bkhetan et al., 2019; Park, 2019) instead of the more accurately deduced pedigree-defined segments/blocks (Alper and Larsen, 2017). The LD-phased DNA sequences are useful but can generate false information that might be misleading in disease association studies (Slatkin, 2008; Tewhey et al., 2011; Alper and Larsen, 2017; Choi et al., 2018; Al Bkhetan et al., 2019). IBD segmental mapping of recent ancestry between individuals in families and populations based on sequence similarity, genotypes, and single-nucleotide polymorphism (SNP) profiles is a newly developed and tested imputation used either with or without LD analysis for inferred haplotype detection (Browning and Browning, 2012; Thompson, 2013; Zhou et al., 2020b).

Major histocompatibility complex disease association studies are most commonly performed at the level of correlations with genotypes, alleles, SNPs (Trowsdale, 2011; Ferreira et al., 2012; Kennedy et al., 2017), microsatellites (Oka et al., 2003; Tamiya et al., 2005; Charfi et al., 2020), and retrotransposon insertion polymorphisms (Dunn et al., 2006; Wang et al., 2017). However, the customary genome-wide association studies (GWAS) of the MHC genomic region are limited severely by the biological complexity of the diseases under investigation and the statistical unreliability and substantial irreproducibility of many analyses that should be examined by also using haplotype genomic structure and haplotypic disease markers (Dawkins et al., 1983; Alper and Larsen, 2017; Kennedy et al., 2017; Lokki and Paakkanen, 2019) including for autoimmune disorders such as systemic sclerosis (Arnett et al., 2010), Graves’ disease (Macel et al., 2001), selective immunoglobulin A deficiency (Ferreira et al., 2012), Parkinson disease (Wissemann et al., 2013), type 1 diabetes (T1D), and celiac disease (Farina et al., 2019).

The main barrier to expanding large-scale haplotype studies at the genomic sequence level in different worldwide populations has been the difficulty of accurately and reliably obtaining long stretches of phased DNA within the MHC and other genomic regions to perform comparative haplomics (O’Neill, 2009). Although next-generation sequencing methods can generate phased DNA and haplotypes (Huang et al., 2017; Choi et al., 2018; Suzuki et al., 2018), much of this is still experimental and relatively too expensive and complicated for most research laboratories to incorporate easily into their current sequencing and genotyping protocols and analytical pipelines. The use of homozygous cell lines is one approach to overcoming the uncertainty of using diploid DNA and the current technical problems of generating phased DNA (Dorak et al., 2006; Horton et al., 2008; Norman et al., 2017). These phased MHC genomic sequences provide representative haplotype panels for better informed large population studies, mapping heterozygous sequence reads (Traherne, 2008; Lam et al., 2013, 2015) and disease associations (Alper and Larsen, 2017; Lokki and Paakkanen, 2019). Although Norman et al. (2017) produced an important database for 95 MHC homozygous cell lines of assembled MHC genomic sequences, their own DNA sequence analyses were limited to describing the multilocus alleles and haplotypes of the HLA classical class I and class II genes, *MUC22* and the structural diversity of *C4* duplications.

Major histocompatibility complex haplotype diversity is driven largely by segmental shuffling and meiotic recombination (Traherne et al., 2006), and this exchange between genomic segments or blocks can be identified by high and low SNP-density XOs at the junctions of different haplotypic blocks (Larsen et al., 2014; Kulski et al., 2021). The analysis of haplotype segmental exchange provides an important insight into IBD due to recent common ancestry for at least 3,400 generations (Traherne et al., 2006), the evolutionary history of ancestral recombinations, and the mechanisms that are involved in generating IBD segment, haplotype, and SNP diversity (Zhou et al., 2020a,b). Therefore, SNP-density XOs between neighboring haplotype blocks are a potential qualitative and quantitative measure of segmental exchanges in the MHC (Traherne et al., 2006; Lam et al., 2013, 2015; Larsen et al., 2014; Kulski et al., 2021) as well as for inferred IBD segments in at least 11 other regions of the human genome (Browning and Browning, 2020; Nait Saada et al., 2020).

Many repeat elements and transposable elements (TEs) that make up > 50% of the human DNA content have contributed to various diseases (Ayarpadikannan and Kim, 2014; Payer et al., 2017; Payer and Burns, 2019), gene regulation and recombination (Moolhuijzen et al., 2010; Myers et al., 2010; Altemose et al., 2017; Chuong et al., 2017) as well as to the duplicated segmental organization of the human and other primate MHC genomic structures (Kulski et al., 1997, 1999a,b, 2000a,b, 2004; Anzai et al., 2003). Because of their mobility, hypermutability, and potential participation in recombination, TEs are integral to molecular drive (Dover, 1993) and together with point mutations, gene conversion (Adamek et al., 2015), and balancing selection (van Oosterhout, 2009), have contributed to generating haplotypic polymorphisms in the MHC class I and class II regions (Andersson et al., 1998; Shi et al., 2014; Kulski et al., 2021). The role of TEs in recombination events is evidenced in part by the structural biallelic Alus, short interspersed nuclear element–VNTR–Alus (SVAs), long terminal repeats (LTRs), and human endogenous retroviruses (HERVs) located either near or within putative recombination hotspots throughout the human genome (Katzourakis et al., 2007; Konkel and Batzer, 2010; Burns and Boeke, 2012; Ayarpadikannan and Kim, 2014; Thomas et al., 2018; Wallace et al., 2018) and the MHC class I and class II genomic regions (Kulski et al., 2011, 2021). In a study of expression quantitative trait loci within the genomic sequences of lymphoblastoid cell lines, Spirito et al. (2019) found that the chromosomal location 6p21.32, which includes the extended MHC class II region from *TNXB* to *DAXX*, was one of the two most enriched genomic regions where structurally polymorphic TEs influenced gene expression.

As part of our previous studies on the importance of TEs as evolutionary and haplotypic markers both in population and comparative sequence analyses, we reported on their role in haplotype shuffling and their linkages to HLA class I alleles in the MHC class I region (Kulski et al., 2021). In this study, we have extended our analysis of haplotype shuffling and the linkages between TE and HLA gene alleles within the MHC class II region of the Norman et al. (2017) sequences to identify and characterize (1) the particular haplotypic linkages between the HLA class II genic and intergenic structurally polymorphic TEs and (2) ancestral SNP-density XO loci in DNA sequence alignments of different haplotype blocks or segments across the ∼1 Mb-extended MHC class II genomic region from the telomeric *PRRT1* gene to the centromeric *COL11A2* gene. We identified a variety of structural bi-allelic TEs that may be useful as lineage markers and confirmed the presence of numerous regions of haplotype exchanges between low and high SNP density XOs at putative ancestral recombination sites that are consistent with and extend the observations of other investigators who have mapped recombination hotspots in the HLA class II region.

We identified 41 structural bi-allelic TE haplotypic markers and confirmed the presence of numerous regions of haplotype exchanges between low and high SNP density XOs at putative ancestral recombination sites that are widely distributed across the ∼1 Mb-extended MHC class II genomic region from the telomeric *PRRT1* gene to the centromeric *COL11A2* gene.

## Materials and Methods

The main sequences and methods used in this study were previously described by Kulski et al. (2021). Essentially, the haplotype data of 95 MHC genomic sequences sequenced and assembled from HLA-homozygous cell lines by Norman et al. (2017) at the National Center for Biotechnology Information (NCBI) BioProject with the accession number PRJEB6763^1^ were downloaded as Fasta files and used for the analyses described later. The other MHC genomic sequences used in haplotype analyses were the GRChr38.p13 (GCF_000001405.39) of the chromosome 6 reference NC_ 000006.12 at the NCBI^2^, Ensembl^3^, University of California, Santa Cruz^4^ browsers and databases, eight human reference haplotypes described by Horton et al. (2008), one chimpanzee sequence of the *MTCO3P1* pseudogene (AC275796.1), four gorilla *MTCO3P1* sequences (AC270181.1, CT025711.1, CT025621.2, AC270177.1), and one orangutan *MTCO3P1* sequence (AC206450.4). All of the Fasta sequences downloaded from the public archives were submitted to the RepeatMasker webserver^5^ for output files of annotated members of the interspersed repetitive DNA families, their locations in the sequence, and their relative similarity or identity in comparison with reference sequences of short interspersed retrotransposable elements (SINEs), long interspersed retrotransposable elements (LINEs), LTRs, HERVs, DNA elements, small RNA, and simple repeats using the Dfam database (3.0) for the repeat sequence comparisons (Hubley et al., 2016).

Norman et al. (2017) provided the alleles for *HLA-DRB1*, *-DRB2*, *-DRB3*, *-DRB4*, and -*DRB5 -DQA1*, *-DQB1*, *-DPA1*, and -*DPB1* for all the 95 cell line sequences shown in Supplementary Table 1. We extracted the sequences and assigned the haplotyped alleles to another seven loci, *HLA-DQA2*, *-DQB2*, *-DOB*, *-DOA*, *-DPB2*, *-DPA3*, and the 660-bp pseudogene *MTCO3P1* in 90 of the 95 cell line sequences by comparing them to the HLA allele sequences in IPD-IMGT/HLA (^6^ Release 3.42.0) and those in GenBank^7^ using the DNA sequence assembly software Sequencher ver.5.0 (Gencode^8^). The new HLA class II alleles are reported here without providing any further information about the novel nucleotide or amino acid differences (Supplementary Table 2). A laboratory identifier number (ID_1 to ID_95) was added to each of the Norman et al. (2017) sequences (Supplementary Tables 1, 2) for ease of identification in comparative sequence analysis. The TE dimorphisms (absence or presence) were easily recognized in each of the RepeatMasker outputs because of their periodic positions within or close proximity of other TE elements and short tandem repeats (STRs). Comparative sequence alignments between two or more sequences to evaluate SNP densities and determine SNP-density XO regions between SNP-poor regions of < 10 SNPs per 100 kb and SNP-rich regions of > 50 SNPs per 100 kb were performed with the web-based MultiPipMaker alignment program^9^ by uploading the Fasta sequence files, a RepeatMasker output file and using the MultiPipMaker setting for single coverage as described by Schwartz et al. (2000) to generate the optimal sequence alignment. SNPs in the alignments were counted twice manually and averaged. Obvious assembly errors, polynucleotides, simple microsatellite repeats, and indels were not counted as SNPs. Also, a series of many adjoining SNPs (e.g., > 5 SNPs in a string of 50 nucleotides) or SNPs within 50 bp of obvious sequencing errors with runs of unspecified nucleotides (Ns) and/or inconsistent long strings of deletions were not counted. The length of sequence alignments usually ranged between 50 and 500 kb depending on (1) the segments targeted for the analysis and the ease of SNP manual counting in the pdf outputs of the nucleotide alignments and/or (2) the length of the percentage identity plot output for reproduction as a convenient and readable image. The targeted sequences were selected and trimmed from the Fasta files previously downloaded from the NCBI BioProject, accession number PRJEB6763. The software program Genetyx ver.20 (GENETYX Co., Tokyo, Japan) was used with the Selector function set to select and trim to obtain the required Fasta file sequences with the genomic sequence target positions guided by those listed in the RepeatMasker output text file. SNP-density plots of selected haplotype sequence alignments were drawn using Microsoft Excel for Mac 2019 from inputs of sequence alignments created by the online MultiPipMaker.

**FIGURE 1:**
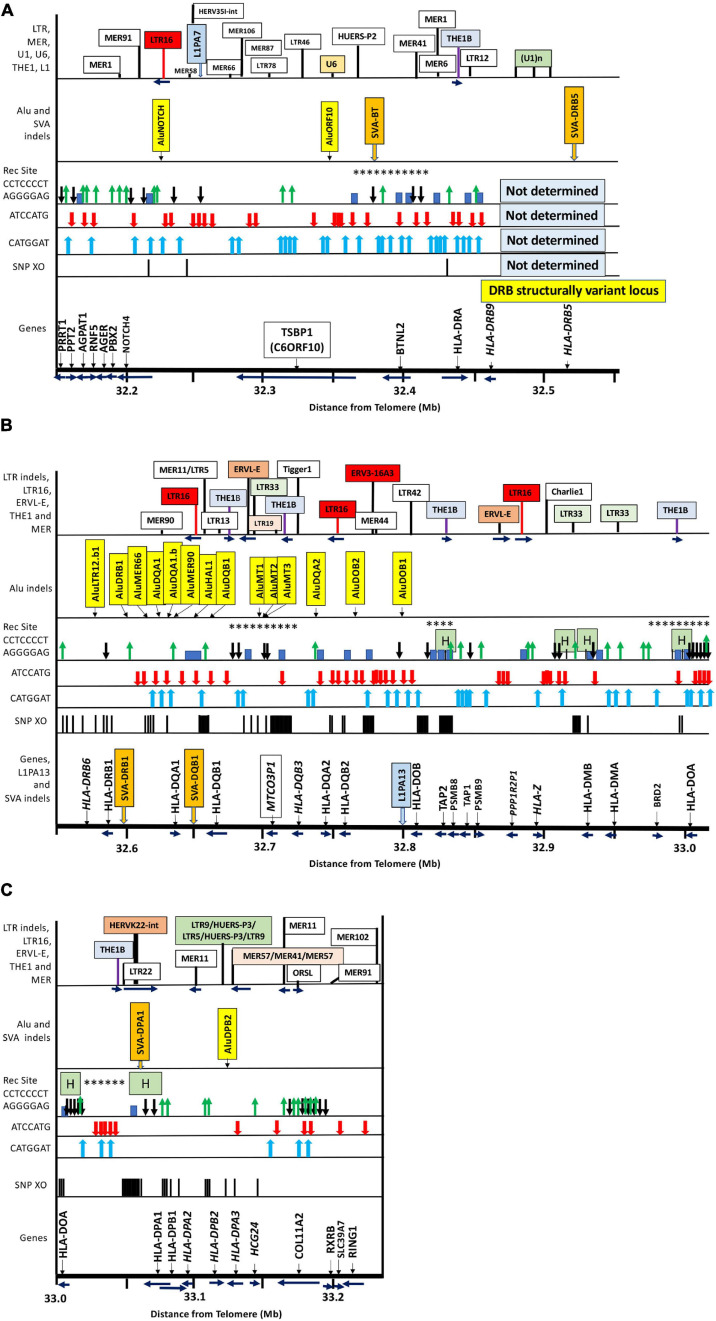
Locations of gene markers (pseudogenes in italics), SNP-density crossover points (haplotype shuffling), recombination sites (Rec Site), PRDM9 partial binding and suppression motifs, Alu and SVA indels, and particular repeat elements used as location tags within ∼1 Mb of MHC class III/class II genomic sequence from *PRRT1* to *RING1* and the nucleotide position 32.150 to 33.22 Mb distance from telomere on chromosome 6 (sequence NC_000006 at NCBI, UCSC, ENSEMBL): **(A)** The MHC class III/II boundary from 32.15 to 32.55 Mb including the Class III genes *PRRT1* to *BTNL2* and the Class II genes, *HLA-DRA* and *HLA-DRB9* and *HLA-DRB5*– within the DRB structural variant locus; **(B)** the MHC class II region from 32.55 to 33.01 Mb with the location of the duplicated HLA class II genes from *HLA-DRB6* to *HLA-DOA*; **(C)** the MHC class II region from 33 to 33.22 Mb with the location of the duplicated HLA class II genes from *HLA-DOA* to *HLA-DPA3* with the extended centromeric region containing the *COL11A2, RXB, SLC3A7* and *RING1* genes. Each Figure A to C contains labeled boxes showing the following comparative items: Genomic position of SNP-density crossover points (SNP XO) indicated by vertical black lines. The genomic position of the PRDM9-suppression sequence motifs *ATCCATG* and *CATGGAT* indicated by red and blue vertical arrows, respectively. The ‘Rec Site’ boxes represent the putative regions of ancestral meiotic recombinations and gene conversions as indicated by the PRDM9 partial binding motif *CCTCCCCT* (black vertical arrow with head down) and its complimentary sequence *AGGGGAG* (green vertical arrow with head up). The blue blocks are putative recombination sites identified by Lam et al. (2013). The H green boxes are ‘hotspots’ identified by Jeffreys et al. (2001), Kauppi et al. (2005), Kong et al. (2010) and Pratto et al. (2014), and highlighted in the NCBI browser (Table 7). The asterix (^∗^) are the meiotic recombination positions identified in sperm studies by Cullen et al. (1997, 2002). The ‘Alu indels’ boxes show the location of the dimorphic Alu listed in Table 3, and the dimorphic SVA are shown in the ‘Alu indels’ boxes for **(A)** and **(C)** and in the ‘Genes’ box for **(B)**. The top boxes of ‘LTR indels’ show the genomic position of selected TE as location tags for orientation and because some of them such as *MER1* and *MER11* harbor PRDM9 motifs or because some such as *LTR16*, *LTR19*, *LTR33* and *THE* sequences have a possible role in recombination initiation and/or suppression.

The “find” option of the Preview v11 software (Apple Inc.) was used to search for the PRDM9 binding motifs *CCTCCCCT/AGGGGAGG* and *ATCCATG/CATGGAT* in MultiPipMaker pdf outputs of the centromeric end of MHC class III and the entire MHC class II region to the *COL11A2* gene (Figure 1) in the trimmed human genomic reference sequence GRChr38.p13 (NC_000006.12). No text wrapping or different formats or layouts of the same sequence were applied in the search.

The T-Coffee multiple sequence alignment tool (Notredame et al., 2000) at EMBL-EBI^10^ was used to submit multiple sequences of *MTCO3P1* in the Fasta format (Supplementary Table 3) with an input maximum file size of 1 Mb resulting in the following outputs of an alignment file in the CLUSTALW (1.83), a simple guide tree or phylogram (dnd format), a neighbor-joining phylogenetic tree without distance corrections (ph format), and a percentage identity matrix (pim format) created by Clustal2.1.

## Results

### Extended Major Histocompatibility Complex Class II Targeted Genomic Region

Figure 1 shows a summary map of the locations of MHC class II gene markers (Genes), SNP-density XO sites, published recombination sites (Rec Site), *ATCCATG* and *CATGGAT* PRDM9 recognition motifs, and dimorphic TE that we identified and analyzed in the extended MHC class II region from the telomeric *PRRT1* gene in the class III region to the centromeric *COL11A2* gene in the class II region on the short arm of chromosome 6, GRCh38.p12 Primary Assembly NC_000006.12; NCBI, UCSC, or ENSEMBL browsers on the Web. The PRDM9 recognition motifs are only for the genomic reference sequence NC_000006.12, and the variations between haplotypes are not shown. Table 1 shows a summary of the types of TE repeats identified by RepeatMasker in the MHC class III genomic region from *PRRT1* to *DRB5* (400 kb) and the MHC class II region from *DRB1* to the *COL11A2* gene (650 kb). Overall, there were ∼1206 TE within 1.050 Mb of a genomic sequence, 474 SINES (353 Alus, 121 MIRs), 383 LINES (260 L1, 110 L2, and 13 L3/CR1), 184 LTR elements, 148 DNA elements, and 17 unclassified elements at 51.3% of the 1,050-kb genomic content. Of the % content of the different family types of TEs, there are relatively fewer SINEs and LINEs and more HERVs and DNA elements in the MHC class II than the class III region. Most of these TEs are inherited from the hominoids (great apes) and fixed in the extended MHC class II region of humans.

**TABLE 1:** Summary of TE repeat sequence families in the extended MHC class II region.

sequences:	*PRRT1* to *DRB5* in MHC class III			*DRB1* to *COL11A2* in MHC class II		
position ref seq-chr6:	32,150,001–32,550,000			32,550,000–33,200,000		
total length:	400,000 bp			650,001 bp		
GC level:	42.8%			42.4%		
bases masked:	223,385 bp (55.9%)			328,250 bp (50.5%)		
	number	bp	%	number	bp	%
SINEs:	214	55,670	13.9	260	63,036	9.7
ALUs	168	48,344	12.1	185	51,556	7.9
MIRs	46	7,326	1.8	75	11,480	1.8
LINEs:	144	107,163	26.8	239	148,782	22.9
LINE1	97	89,411	22.4	162	130,405	20.1
LINE2	45	17,417	4.4	65	16,191	2.5
L3/CR1	2	335	0.2	11	1,915	0.3
LTR elements:	64	40,719	10.3	121	75,934	11.7
ERVL	9	2,758	0.7	30	10,402	1.6
ERVL-MaLRs	12	6,210	1.6	50	19,878	3.1
ERV_class I	39	26,240	6.6	26	22,789	3.5
ERV_class II	3	5,389	1.4	10	21,582	3.3
DNA elements:	53	10,175	2.5	95	27,078	4.2
hAT-Charlie	26	4,312	1.1	41	11,847	1.8
TcMar-Tigger	11	4,303	1.1	24	9,659	1.5
Unclassified:	7	3,873	1.0	10	5,085	1.0
Total interspersed repeats:		217,600	54.4		319,915	49.2
Small RNA:	7	497	0.2	14	949	0.2
Simple repeats:	85	3,842	1.0	15	6,012	0.9
Low complexity:	19	1,461	0.4	24	1,374	0.2

*Major histocompatibility complex class III and class II regions are shown in Figure 1.*

### Major Histocompatibility Complex Class II Haplotype Sequences

Supplementary Table 1 shows a comparative analysis of HLA class II gene alleles at 14 loci, including the pseudogene *MTCO3P1* from *HLA-DR* to *HLA-DP* and haplotype (allele) shuffling between genomic sequences (Hap ID) obtained from 95 homozygous cell lines (Norman et al., 2017). There are two to eight identical long-range haplotypes from *DR* to *DOA* loci that share the same combinations of alleles. For example, there are eight cell lines with the 8-locus haplotype *DRB1^∗^04/DQA1^∗^03/DQB1^∗^03/MTCO3P1^∗^08/DQA2^∗^01/DQB2^∗^ 01/DOB^∗^01/DOA^∗^01*. Most of the other haplotypes have an obvious transition between gene alleles at least at one of the 10 loci between *HLA-DRB1* and *HLA-DPA3*. Table 2 shows 26 distinct *DRB1/DQA1/DQB1/MTCO3P1/DQA2/DQB2* haplotype lineages in 87 of the Norman et al. (2017) sequences. The two most common haplotypes were 10 *DRB1^∗^03/DQA1^∗^05/DQB1^∗^02/MTCO3P1^∗^01/DQA2^∗^01/DQB2 ^∗^01* and eight *DRB1^∗^04/DQA1^∗^03/DQB1^∗^03/MTCO3P1^∗^08/DQA2^∗^01/DQB2^∗^01*. Various allelic transitions have occurred in a location between the *HLA-DQB1* and *MTCO3P1* loci. For example, there are four different haplotype linkages between the *HLA-DQB1* alleles and *MTCO3P1^∗^03* and seven between HLA-*DRB1* and *MTCO3P1^∗^03*.

**TABLE 2:** Twenty-six distinct *DRB1-DQA1-DQB1-MTCO3P1-DQA2-DQB2* haplotype lineages in 87 of the Norman et al. (2017) sequences.

HAP ID #	*HLA-DRB1*	*HLA-DQA1*	*HLA-DQB1*	*MTCO3P1*	*HLA-DQA2*	*HLA-DQB2*	Number	Potential Disease Phenotypes
1	DRB1*09	DQA1*03	DQB1*03	MTCO3P1*03	DQA2*01	DQB2*01	2	AH 46.1
2	DRB1*07	DQA1*02	DQB1*02	MTCO3P1*04	DQA2*01	DQB2*01	7	AH 47.1
3	DRB1*07	DQA1*02	DQB1*03	MTCO3P1*03	DQA2*01	DQB2*01	2	
4	DRB1*04	DQA1*03	DQB1*03	MTCO3P1*08	DQA2*01	DQB2*01	8	T1D
5	DRB1*04	DQA1*03	DQB1*03	MTCO3P1*10	DQA2*01	DQB2*01	1	T1D
6	DRB1*04	DQA1*03	DQB1*03	MTCO3P1*03	DQA2*01	DQB2*01	3	T1D
7	DRB1*04	DQA1*03	DQB1*04	MTCO3P1*07	DQA2*01	DQB2*01	6	
8	DRB1*08	DQA1*06	DQB1*03	MTCO3P1*03	DQA2*01	DQB2*01	1	
9	DRB1*08	DQA1*01	DQB1*06	MTCO3P1*09	DQA2*01	DQB2*01	1	
10	DRB1*11	DQA1*05	DQB1*03	MTCO3P1*03	DQA2*01	DQB2*01	6	Scleroderma
11	DRB1*11	DQA1*01	DQB1*05	MTCO3P1*03	DQA2*01	DQB2*01	1	
12	DRB1*11	DQA1*01	DQB1*06	MTCO3P1*02	DQA2*01	DQB2*01	1	
13	DRB1*12	DQA1*05	DQB1*03	GAP	DQA2*01	DQB2*01	1	
14	DRB1*13	DQA1*01	DQB1*06	MTCO3P1*05	DQA2*01	DQB2*01	4	
15	DRB1*13	DQA1*01	DQB1*06	MTCO3P1*02	DQA2*01	DQB2*01	4	
16	DRB1*14	DQA1*01	DQB1*05	MTCO3P1*06	DQA2*01	DQB2*01	3	MG
17	DRB1*14	DQA1*01	DQB1*06	GAP	DQA2*01	DQB2*01	1	
18	DRB1*14	DQA1*05	DQB1*03	GAP	DQA2*01	DQB2*01	1	
19	DRB1*03	DQA1*04	DQB1*04	MTCO3P1*07	DQA2*01	DQB2*01	1	AH 42.1
20	DRB1*03	DQA1*05	DQB1*02	MTCO3P1*01	DQA2*01	DQB2*01	10	8.1 AH, MAD, T1D, CD, GD
21	DRB1*01	DQA1*01	DQB1*05	MTCO3P1*09	DQA2*01	DQB2*01	5	
22	DRB1*15	DQA1*01	DQB1*06	MTCO3P1*02	DQA2*01	DQB2*01	7	7.1AH, MS, PD, MNCs
23	DRB1*15	DQA1*01	DQB1*06	MTCO3P1*03	Not included	Not included	1	
24	DRB1*15	DQA1*01	DQB1*06	MTCO3P1*04	DQA2*01	DQB2*01	3	
25	DRB1*16	DQA1*01	DQB1*05	MTCO3P1*03	DQA2*01	DQB2*01	5	MG
26	DRB1*16	DQA1*05	DQB1*03	MTCO3P1*03	DQA2*01	DQB2*01	2	
						Total	87	

*AH, ancestral haplotype; T1D, type 1 diabetes; CD, celiac disease; MG, Myasthenia gravis; MAD, multiple autoimmune disease; GD, Graves’ disease, MS, multiple sclerosis; MNCs, multiple neurological conditions; PD, Parkinson’s disease.Dawkins et al. (1999), Macel et al. (2001), Dorak et al. (2006), Arnett et al. (2010), Wissemann et al. (2013), Farina et al. (2019), Lokki and Paakkanen (2019).*

The alleles for the 660-bp *MTCO3P1* pseudogene were determined in 84 of the Norman et al. (2017) sequences because there are at least 19 SNP-density XOs near its locus (Figure 1). Ten alleles (Supplementary Table 3) were determined for 84 *MTCO3P1* sequences, and these were all haplotypic (Supplementary Table 2). *MTCO3P1* haplospecificities were observed between *MTCO3P1^∗^01* and *DRB1^∗^03/DQA1^∗^05/DQB1^∗^02*; *MTCO3P1^∗^06* and *DRB1^∗^14/DQA1^∗^01:04/DQB1^∗^05:03*; and *MTCO3P1^∗^09* and *DRB1^∗^01/DQA1^∗^01/DQB1^∗^05*. The haplotype *DRB1^∗^13/DQA1^∗^01/DQB1^∗^06* was linked with either *MTCO3P1^∗^02* or *MTCO3P1^∗^05*. On the other hand, *MTCO3P1^∗^03* in 22 sequences was linked with *DRB1^∗^04, DRB1^∗^07*, *DRB1^∗^08*, *DRB1^∗^09*, *DRB1^∗^11*, *DRB1^∗^15*, and *DRB1^∗^16*. The linkage of *MTCO3P1^∗^03* with seven different *HLA-DRB1* allelic lineages suggests that this particular *MTCO3P1* allele was transmitted *via* many ancestral recombination events *via* haplotype shuffling and is probably the oldest of the 10 alleles. Some of the other *MTCO3P1* alleles were also linked with multiple *HLA-DRB1* alleles: *MTCO3P1^∗^02* with *DRB1^∗^13* and *DRB1^∗^15*; *MTCO3P1^∗^04* with *DRB1^∗^07* and *DRB1^∗^15*, *MTCO3P1^∗^07* with *DRB1^∗^03*, *DRB1^∗^04* and *DRB1^∗^08*; and *MTCO3P1^∗^09* with *DRB1^∗^01* and *DRB1^∗^08* (Table 2).

**FIGURE 2:**
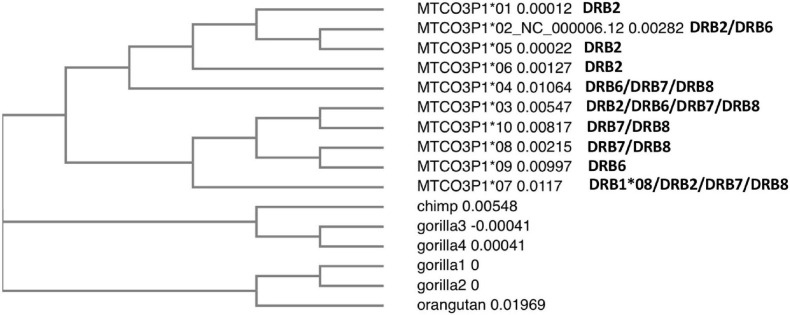
Phylogenetic tree of the 10 human *MTCO3P1* allele sequences that were aligned by clustalW and compared to the *MTCO3P1* sequences of a chimpanzee, four gorillas, and an orangutan.

Figure 2 shows a phylogenetic tree of the 10 human *MTCO3P1* allele sequences aligned and compared with the *MTCO3P1* sequences of a chimpanzee, four gorillas, and an orangutan. Two human *MTCO3P1* sequence clusters separated from the ape sequences, suggesting that the *MTCO3P1* alleles are human-specific. One cluster of four human *MTCO3P1* alleles 01, 02, 05, and 06 that are mostly associated with the *DRB2* (*DR52* haplotype) lineage separated from the *MTCO3P1^∗^04* allele branch that was linked with the *DRB6/DRB7/DRB8* lineages. This separation suggests that the *DRB2* (*DR52* haplotype) lineage had branched from the *DRB6* (*DR1/DR51* haplotype) and *DRB7/DRB8* (*DR53* haplotype) lineages as previously proposed by Andersson (1998). The other cluster of five human *MTCO3P1* alleles has *MTCO3P1^∗^09* linked with the *DRB6* (*DR1* haplotype) and the *HLA-DRB1^∗^08* lineage; *MTCO3P1^∗^08* and *MTCO3P1^∗^10* linked to the *DRB7/DRB8* (*DR53* haplotype) lineage; and *MTCO3P1^∗^03* and *MTCO3P1^∗^07* linked with various other *DR* loci. This suggests that numerous recombination events had occurred after the proposed initial evolutionary separation between the *DR53* haplotype (*DRB7/DRB8*) and haplotype lineages of *DR52* (*DRB2*), *DR51* (*DRB5* and *DRB6*), *DR1* (*DRB6*), and *DR8* (*DRB1^∗^08* allelic lineage) more than 65 million years ago (Andersson, 1998; Andersson et al., 1998).

### Class II Dimorphic Transposable Elements and Their Linkages With Human Leukocyte Antigen Class II Gene Alleles

Eighty-nine of the 95 human MHC haplotypes were examined for the presence of dimorphic TE represented by Alus, SVAs, LTRs, and HERVs in RepeatMasker outputs of the interspersed repetitive DNA families; their locations in the sequence and their relative similarity were compared with reference sequences of SINEs, LINEs, LTRs, HERVs, DNA elements, small RNA, and simple repeats. The five class II polymorphic Alu insertions, *AluORF10*, *AluDRB1*, *AluDQA1*, *AluDQA2*, and *AluDPB2*, were easily identified within the RepeatMasker outputs on the basis of their location and flanking sequences as previously described (Kulski et al., 2010). Of the 44 TE indels (absent or present) examined in the present analysis (Table 3), two were monomorphic (*SVA-BT*, *LTR16*) and appear to have been fixed in the human genome at least before the divergence of humans and chimpanzees (data not shown). One of the indels, TE-ID#44 that is located in the region between *HLA-DQB1* and *MTCO3P1*, is a 7-kb mosaic composed of at least 10 other TE family members, including *LTR9*, *AluSx*, *HUERS-P3*, *MER51*, *MER58*, *MER61*, *MER63*, and *LTR33* (Table 4). The *MLT1E*, *MSTC*, and *AluSx* TE sequences within the 7-kb insertion were deleted separately from some of the other ID #44 insertion sequences. Moreover, the 7-kb insertion (with the *LTR9* and *LTR33* sequences) was linked haplotypically to all 22 *MTCO3P1^∗^03*, six *HLA-DQB1^∗^0502*, 15 of 16 *HLA-DQB1^∗^03:01*, and one of five *HLA-DQB1^∗^06* sequences (Supplementary Table 4). Although any combination of these particular TEs could be used as genetic markers for these haplotypes, caution is required in constructing primers and probes because the *MLT1E*, *MSTC*, and *AluSx* TE sequences are duplicated in a region between *HLA-DQB2* and *HLA-DOB* (Table 4), and a relatively full-length *LTR9-AluSx-HUERS-P3* sequence is inserted between exons 2 and 3 within the *HLA-DPB2* pseudogene sequence (Supplementary Figure 1).

**TABLE 3:** Dimorphic TE (indels, absent or present) analyzed in this study.

TE-ID #	Retroelement	Nearest Flanking (/) Gene(s)	Location within Genome Reference Ch38/hg38, Chr 6 (strand)	Distance between TE and gene loci, bp			NCBI dbVar Curated Common SVs	1000 genomes SVs DGVa estd214, estd219
				*DRB1*	*DQA1*	*DQB1*		
1	*AluORF10*	*C6orf10*	32346003-32346314 (−)	232,455	291,403	313,153	nssv16196990	esv3844276
2	*AluLTR12.DRB5*	*DRB5*	32494420-32494692 (−)	84,077	142,714	164,775		
3	*AluLTR12.DRB1*	3′ of *DRB1*	32579821-32580132 (+)	1,052	57,274	79,335		
4	*AluDRB1*	5′ of *DRB1*	32603572-32603844 (+)	13,712	33,834	55,623	nssv16191854	esv3608598, esv3844294
5	*AluMER66*	5′ of *DQA1*	∼32621979 (+)	32,131	15,427	37,448		
6	*AluDQA1(.a)*	5′ of *DQA1*	32625934-32626239 (−)	36,086	11,472	33,228	nssv16185199	
7	*AluDQA1(B)*	5′ of *DQA1*	32629720-32629999 (−)	39,872	7,407	29,468		
8	*AluMER90.DQA1*	5′ of *DQA1*	32634001-32634251 (+)	44,153	3,155	25,216	nssv16196737	
9	*AluSx.SV1*	3′ of *DQA1*	32647187-32647484 (+)	57,339	3,535	11,983	nssv16197586	esv3608604
10	*AluHAL1ME*	3′ of *DQA1*	32652010-32652295 (+)	62,162	14,604	7,172	nssv16185138	
11	*AluDQB1*	5′ of *DQB1*	∼32663914 (+)	74,066	20,244	4,447	nssv16191333	
12	*AluTHE1A.DQB1*	3′ of *DQB1*	32673367-32673677 (+)	83,520	29,697	4,985		
13	*AluMT1*	3′ of *MTCO3P1*	∼32689573/32690722 (−)	99,725	45,902	21,190		
14	*AluMT2*	5′ of *MTCO3P1*	32699625-32699910 (+)	109,777	55,954	31,242		
15	*AluMT3*	5′ of *MTCO3P1*	32703983-32704299 (+)	114,135	60,312	35,600		
16	*AluDQA2*	5′ of *DQA2*	∼32740293 (+)	150,445	96,622	71,910		
17	*AluDOB2*	3′ of *DOB*	32781456-32781764 (−)	191,608	137,785	113,073	nssv16187057	esv3608614
18	*AluDOB1*	3′ of *DOB*	∼32804316 (−)	215,818	161,995	137,283		
19	*AluDPB2*	5′ *DPB2*	33125647-33125965 (+)	535,799	481,976	457,264	nssv16201287	
20	*AluSc8-AluJb*	*HCG24/COL11A2*	33158148-33159074	568,300	514,477	489,765	nssv16192244	
21	*SVA-BTN*	*C6orf10/BTNL2*	32384189-32386122 (−)	194,580	251,284	273,345	monomorphic	
22	*SVA-DRB4/HERV9*	*DRB4*	DBB:3800882 3802512 (−)	5,049	71,597	89,567		
23	*SVA-DRB5*	5′ of *DRB5*	32531532-32532999 (+)	45,770	105,874	126,468	nssv16197894	
24	*SVA-DRB1*	5′ of *DRB1*	32594193-32596780 (−)	4,345	40,626	62,687	nssv16196260	
25	*SVA-DQB1* fragmented	3′ of *DQB1*	∼32653260 (−)	63,412	9,589	6,207		
26	*SVA-DPA1*	3′ of *DPA1*	33058946-33060797 (−)	469,098	415,275	390,563	nssv16182953	esv3608619
27	*SVA-DAXX (harlequin)*	5′ of *DAXX*	33357412-33358020 (−)	767,564	713,741	689,029		
28	*SVA-ZBTB9 (cheshire)*	3′ of *ZBTB9*	33485065-33486622 (−)	895,217	841,394	816,682		
29	*LTR22-DRB7/8*	*DRB7/DRB8*	DBB:3786680-3787163 (+)	21,578	87,443	21,578		
30	*LTR14-DRB1*	3′ of *DRB5*	32512830-32513385 (+)	47,237	124,021	146,082		
31	*LTR3-DRB3/5*		32559719-32567345 (−)	11,424	70,061	92,122		
32	*LTR12-DRB6*	*DRB5/DRB6*	32547022-32548487 (−)	30,282	88,919	110,980	nssv16188969	
33	*LTR12/AluY-DRB1*	*DRB1*	32578751-32581032 (−)	595	57,206	78,291	nssv16194730/	
33	*LTR12/AluY-DRB1*						nssv16195291	
34	*MER11-DRB1*	*DRB1*	∼32586373 (+)	3,475	51,033	73,094		
35	*MER90/MLT2/MER90*	5′ of *DQA1*	32634949-32635931 (−/−/−)	45,101	1,475	23,536		
36	*LTR16-DQA1/DQB1*	*DQA1/-DQB1*	32652876-32653193 (−)	63,028	9,205	6,274		
37	*MER11-DQB1*	3′ of *DQB1*	32655746-32656815 (−)	65,898	12,075	2,652	nssv16194796	esv3608605
38	*LTR5.DQB1*	3′ of *DQB1*	32657125-32658083 (−)	62,277	13,454	2,342	nssv16192810	esv3608606
39	*LTR13-DQB1*	5′ of *DQB1*	∼32667893 (+)	78,045	24,222	490		
40	*L1PA10-DQB1*	5′ of *DQB1*	32674360-32677391 (2 +)	84,512	30,689	5,977	nssv16197816	esv3608607
41	*LTR5-L1PA10-DQB1*	5′ of *DQB1*	∼32676160 (2 +)	86,512	32,689	7,977		
42	*LHS1/Tig4-MT*	*MTCO3P1/DQB3*	32708859-32709255 (−)	119,011	65,188	40,476	nssv16189788	esv3844306
43	*LTR42-DOB*	*HLA-DOB*	32811165-32811646 (+)	221,317	167,494	142,782	nssv16198930	esv3844312
44	*Indel Region A*	*DQB1/MTCO3P1*	∼32687972-32688307	98,124	44,301	19,589		

*UCSC genome browser at https://genome.ucsc.edu was the source of the gene loci positions for HLA-DRB1 (6:32,578,769–32,589,848), HLA-DQA1 (6:32,637,406–32,643,671), and HLA-DQB1 (6:32,659,467–32,668,383) and listed TE positions in the table. NCBI dbVar: https://www.ncbi.nlm.nih.gov/dbvar/; 1,000 genomes SVs: - DGVa: https://www.ebi.ac.uk/dgva/. Dimorphic TEs are structural variants (SVs) or indels. TE-ID #20 noted but not analyzed (insufficient samples).Kulski et al. (2010), The 1000 Genomes Project Consortium et al. (2015a, b), Lee et al. (2020).*

**TABLE 4:** A list of TEs flanking or within (A) a 7-kb indel (boxed) located between *HLA-DQB1* and the *MTCO3P1* pseudogene compared with the TEs in (B) a partially duplicated region located between *HLA-DQB2 and HLA-DOB.*

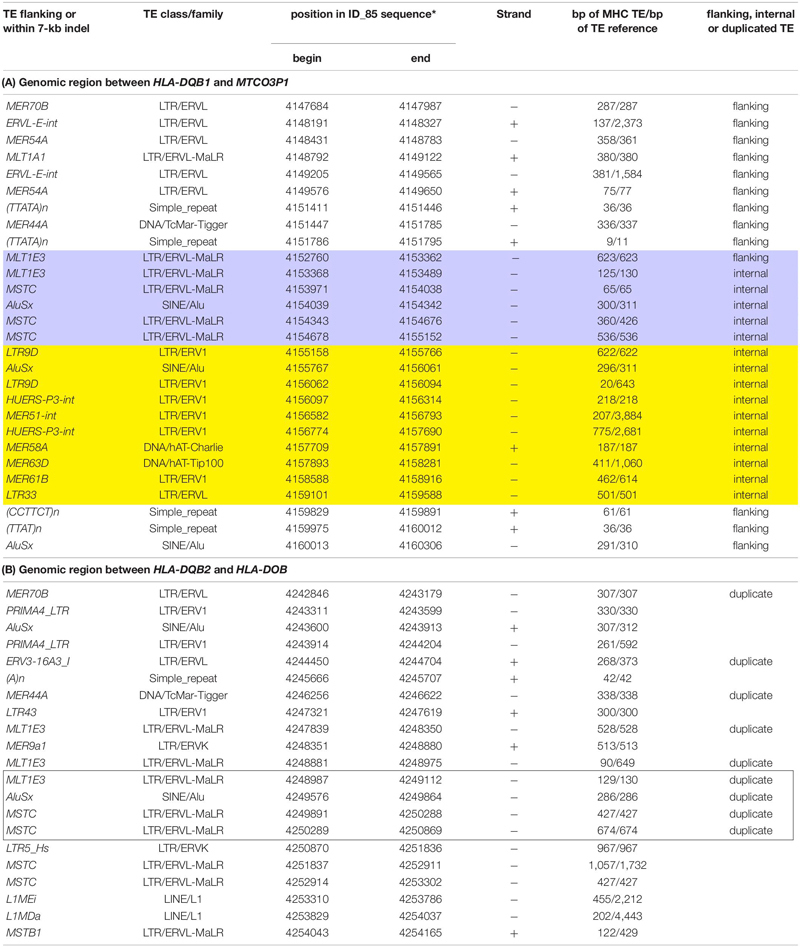

*MCF cell line contains the ID_85 sequence. The 7-kb sequence is deleted in the genome GRChr38.p13 reference sequence. The yellow and blue boxes show the TEs within the 7-kb indel, which represents TE ID_#44 in Supplementary Table 4. The blue box shows the TEs that are also present within the boxed duplicated region (B).

Supplementary Table 4 shows the dimorphic TE linkages with each other and the HLA class II loci, including the *MTCO3P1* pseudogene alleles in the 89 haplotype sequences. Supplementary Table 5 provides a summary of 148 percentage linkage counts between some of the dimorphic TE, the HLA class II gene alleles, and the *MTCO3P1* alleles. Many of these dimorphic TE insertions are haplotypic, but four of the dimorphic Alu insertions appear to be haplospecific: *AluMER66^∗^2* and *HLA-DRB1^∗^01:01:01*, *AluHAL^∗^2* and *DQA1^∗^01*, *AluDQB1^∗^2* and *HLA-DQB1^∗^02*, and the *AluMT1^∗^2* insertion within the *DRB1^∗^07/DQA1^∗^02/DQB1^∗^02/MTCO3P1^∗^04* haplotype. In addition, 10 of the 17 *AluDQB1* insertions are also linked to all 10 sequences with the 8.1 Ancestral haplotype (*DRB1^∗^03:01:01:01/DQA1^∗^05:01:01:02//DQB1^∗^02:01:01/MTCO 3P1^∗^01*).

The number of different MHC class II haplotypes for the Norman et al. (2017) DNA sequences can vary markedly depending on what linkage markers, alleles, and genomic distances are used for the haplotype counts. For example, we counted 29 distinct haplotypes using 14 loci covering a distance of 128,187 bp from the *HLA-DRB1* gene to the *MTCO3P1* pseudogene for 89 sequences, including nine Alu and two LTR indels (LTR13 and LTR33) and the allelic lineages of *HLA-DRB1*, *HLA-DQA1*, *HLA-DQB1*, and *MTCO3P1*. On the other hand, the number increased to 53 different haplotypes simply by extending the distance of the sequence coverage a further 100,000 bp toward *HLA-DOB* with the addition of another five loci, four for Alu indels and one for the *LTR42* indel located near the *HLA-DOB* gene (Supplementary Table 6). In comparison, we counted 26 distinct six-loci *DRB1/DQA1/DQB1/MTCO3P1/DQA2/DQB2* haplotype/allelic lineages in 87 sequences (Table 2). Table 5 shows the six *AluDOB2/AluDOB1/LTR42.DOB* haplotypes in 84 sequences, and Table 6 provides 11 HLA-*DPB2/AluDPB2/HLA-DPA3* haplotypes and the number for each combination in 72 sequences.

**TABLE 5:** *AluDOB2/AluDOB1/LTR42.DOB* haplotypes.

HAP ID	*AluDOB2* allele	*AluDOB1* allele	*LTR42.DOB* allele	Number of haplotypes
hap 1	absent	absent	absent	34
hap 2	absent	absent	present	2
hap 3	absent	present	absent	21
hap 4	present	absent	absent	1
hap 5	present	present	absent	1
hap 6	present	absent	present	25

**TABLE 6:** *HLA-DPB2/AluDPB2/HLA-DPA3* haplotypes.

Hap ID	Haplotype			Number of sequences
	*HLA-DPB2*	*AluDPB2*	*HLA-DPA3*	
hap 1	DPB2*01:01:01	absent	DPA3*01	1
hap 2	DPB2*01:01:01	absent	DPA3*02	1
hap 3	DPB2*01:01:01	absent	DPA3*03	15
hap 4	DPB2*01:01:02	absent	DPA3*02	15
hap 5	DPB2*01:01:02	absent	DPA3*03	1
hap 6	DPB2*03:01:01	absent	DPA3*01	7
hap 7	DPB2*03:01:01	absent	DPA3*03	1
hap 8	DPB2*03:01:01	present	DPA3*01	15
hap 9	DPB2*03:01:01	present	DPA3*03	11
hap 10	GAP	present	DPA3*01	2
hap 11	GAP	present	DPA3*03	3

Because there were numerous gaps and various sequence rearrangements in the *DR* haplotype region between *HLA-DRA* and *HLA-DRB1*, even within the same haplotypes, we could not identify with any confidence the correct positions of the TE indels relative to each other and the *HLA-DR* genes. Instead, we simply counted the numbers for the presence of nine particular TE indels for each haplotype sequence (Supplementary Table 7). The TE counts in 89 sequences were for *U1* (300 counts), *LTR12* (212), *HERV9-LTR12* (139), *LTR43* (42), *LTR5* (41), *MER77* (26), *LTR22* (24), *LTR14* (21), and *LTR14-HERVK14* (8). The TE associated with particular *DR* haplotypes based on the eight haplotype sequences of Horton et al. (2008) is shown in Supplementary Table 8.

### Haplotype Shuffling and Single-Nucleotide Polymorphism-Density Crossovers

Supplementary Tables 1, 2, 4 show numerous gene allele XOs between various shuffling haplotypes across the MHC class II region from the *HLA-DRB1* locus to the *HLA-DP* locus. Sequence alignment comparisons were performed between various MHC class II genomic sequences to locate the site of the last identifiable SNP at the junction between a SNP-rich and a SNP-poor block. Table 7 presents a summary of the number of sequence comparisons and the number of SNP-density XO sites detected. Supplementary Table 9 lists the 171 XOs at 98 unique SNP-density XO nucleotide positions identified from *HLA-DRB6* to *HLA-DPB1* in 121 paired-sequence alignments of different haplotypes. The 98 SNP-density XO positions relative to HLA class II genes and previously identified recombination sites are shown in Figure 1. Most XOs occurred within or between various TE elements, but some were also within *HLA-DRB1*, *-DQB2*, *-DOB*, *-DMB*, *-DOA*, and *-DPA3* gene sequences. In most cases, the XOs were identified in locations between the different gene loci. Fourteen of 98 unique SNP XO sites were identified in the region between the pseudogenes *MTCO3P1* and *HLA-DQB3*, and five XOs were in locations between *HLA-DQB1* and *MTCO3P1*. Thus, 19 XOs were in the vicinity of *MTCO3P1*, compared with 11 XOs in the vicinity of *TAP2* and *PSMB8*, 7 XOs between *HLA-DQB2* and *HLA-DOB*, and 3 XOs between *DOB* and *TAP2*. Also, 13 XOs were found in locations between *HLA-DOA* and *HLA-DPA1* mostly within the *HERVK22* sequence and one within the *SVA-DPA1* indel. Of the 121 sequence alignments with XOs, 81 had a single XO, 31 had 2 XO sites, 8 had 3 XO sites, and 1 (6_ PGF v 73_SPL) had 4 XO sites.

**TABLE 7:** Number of haplotype pair comparisons and SNP-density XO sites detected.

Number of haplotype pairs analyzed	136
Total number of XOs identified	171
Number of single XOs per haplotype pair	81
Number of double XOs per haplotype pair	31
Number of triple XOs per haplotype pair	8
Number of quadruple XOs per haplotype pair	1
Number of haplotype pairs with no XOs	4
Number of haplotype pair comparisons for Figure 3	11
Number of unique XO sites from *HLA-DRB6* to *COL11A2*	98

*Summary counts taken from Supplementary Table 8.*

The longest SNP-free alignments in the 121 comparisons between different haplotype pairs were from:

(1) *HLA-DRB1* to *HLA-DOA* (439–466 kb) for the sequence comparisons between 9-WT47 and 13-SLE005 (*DRB1^∗^13:02/DQA1^∗^01:02/DQB1^∗^06:04/MTCO3P1 ^∗^05*), and 64-YAR and 48-ISH3 (*DRB1^∗^04/DQA1^∗^03:01/DQB1^∗^03:02/MTCO3P1^∗^08*),

(2) *HLA-DRB1* to *3’HLA-DPA1* (460 kb) between 59-AZH and 38-CALEGORO (*DRB1^∗^16:01/DQA1^∗^01;02/DQB1^∗^05:02/MTCO3P1^∗^05*,

(3) *HLA-DRB1* to telomeric (3’) of *HLA-DOA* (408 kb) between 19-LO541265 and 16-PF04015 (*DRB1^∗^03:01/DQA1^∗^05:01/DQB1^∗^02:01/MTCO3P1^∗^01*).

No SNP-density XOs were detected in four paired sequence alignments between the same haplotypes: 51_QBL v 90_LD2B; 62_AKIBA v 93_KAWASAKI; 37_DBB v 58_BEI; and 15_QBL v 26_DUCAF (Supplementary Tables 4, 9).

**FIGURE 3:**
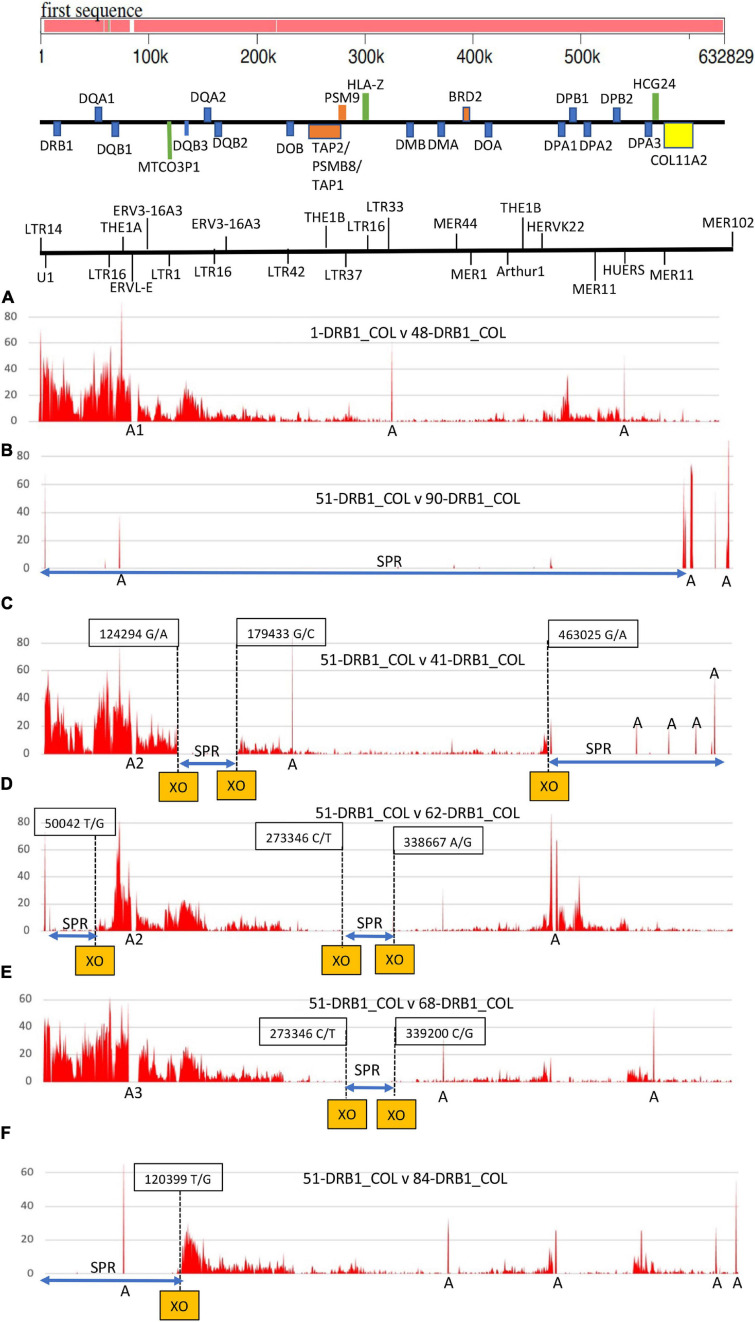
Single-nucleotide polymorphism (SNP) density plots of six pairs of MHC class II haplotypes from *HLA-DRB1* to *COL11A2*. The genomic region and distances for the haplotype sequences are indicated at the top of the Figure with a gene map and an abbreviated TE map highlighting *U1* and a few LTR, MER and HERV elements. The six SNP plots are comparisons between **(A)** *1_ DRB1^∗^01:01/DQA1^∗^01:01/DQB1^∗^05:01:01/MTCO3P1^∗^09* versus *48_ DRB1^∗^04:06/DPA1^∗^02:02/MTCO3P1^∗^08*; and then *51_DRB1^∗^15:01:01:01/DQA1^∗^01:02/DQB1^∗^06:02/MTCO3P1^∗^02* versus **(B)** *90_ DRB1^∗^15:01/DQA1^∗^01:02/DQB1^∗^06:02/MTCO3P1^∗^02*, **(C)** *41_ DRB1^∗^04:01/DQA1^∗^03:01/DQB1^∗^03:02/MTCO3P1^∗^08*, **(D)** *62_ DRB1^∗^15:02/DQA1^∗^01:03/DQB1^∗^06:01/MTCO3P1^∗^04*, **(E)** 68_ *DRB1^∗^11:03/DPA1^∗^01:03/DPB1^∗^04:02/MTCO3P1^∗^03*, and **(F)** 84_ *DRB1^∗^15:01:01/DQA1^∗^01:02:01/DQB1^∗^06:02/MTCO3P1^∗^02*. The Y-axis presents the number of SNPs per 500 nucleotides (window size). The X-axis shows the SNP positions (SNPs/500 nucleotides) in a block of 632829 nucleotides from *HLA-DRB1* to *COL11A2* **(A–F)**. The letter ‘A’ along the X-axis marks regions of sequence gaps, poor assembly, inversions or long runs of unspecified nucleotides. ‘A1’ is the position of a *MER11/LTR5* indel (TE ID #37 and #38) between 76999 and 79305; ‘A2’ is the position of a 4562-bp *LTR5/L1PA10* indel (TE ID #41); and A3 is the position of a 9324-bp indel at 77368-86691 that includes the presence or absence of *MER4* and *L1PA10* together with the *THE1A-AluY-THE1A* and *LTR5* (see TE IDs #12, #40 and #41 in Table 3 and Supplementary Table 3). The dashed vertical lines mark the SNP-density crossover (XO) points between haplotype pairs. The boxed number is the XO sequence position for sequence ID_51.

SNP-density plots across the entire class II region for the same haplotype pair and five different haplotypes are shown in Figure 3. Two different SNP-rich haplotypes (ID_1 v ID_48) produced a typical SNP density profile of four major peaks and troughs decreasing in height and intensity from the *HLA-DRB1* gene over 600 kb to the *COL11A2* gene. The two highest SNP-density peaks were in the region of the *HLA-DRB1* gene and the *DQA1/DQB1* gene cluster and then smaller peaks in the regions of *MTCO3P* to *DQA2* and separately in the *HLA-DP* cluster. By comparison, the SNP density was very low in a 191.5-kb genomic region between *HLA-DOB* and *HLA-DOA* that included the *TAP2/PSMB8/TAP1/PSMB9* genes, *HLA-DMB*, *HLA-DMA*, and *BRD2*. On the other hand, the SNP plot between the same haplotype sequences (ID_51 v ID_90) produced no peaks or troughs because there were essentially no SNPs (SNP-poor) to be counted over 600 kb of sequence. Figure 3B shows a few narrow peaks labeled “A” that are assembly and alignment errors, inversions, and/or long runs of unspecified nucleotides. The other four SNP density plots (C) to (F) in Figure 3 show discernible SNP-density XO points at the junctions of SNP-poor and SNP-rich segments in the alignments between different haplotypes (Supplementary Table 4); three XOs in (C) and (D), two XO in (E), and one XO in (F).

### PRDM9 Recombination Motifs Across ∼1 Mb of Genomic Sequence From *PRRT1* to *COL11A2*

Myers et al. (2005, 2010) identified a consensus PRDM9 binding motif *CCTCCC[CT]AGCCA[CT]* associated with recombination hotspots and genomic instability in humans, whereas Altemose et al. (2017) found an *ATCCATG* motif that might inhibit recombination and that they considered was one of the most common non-PRDM9 recombination-influencing motifs. Table 8 shows the meiotic recombination sites and genomic positions within the MHC class II region annotated by the NCBI Genome Data Viewer^11^. Most of these sites have the nucleotide motif with similarity to the predicted 13-mer PRDM9 binding motif *CCNCCNTNNCCNC* (16 nt).

**TABLE 8:** Meiotic recombination sites within the MHC class II region annotated by the NCBI Genome Data Viewer.

Meiotic Recombination Site (size nt)	GeneID	Nearest Gene	TEs at site
32835539-32837158 (1620)	107648851	*TAP2*	GA-rich repeats/*Tigger3a/MER96*
		*LOC107648851*	
32836099-32837694 (1596)	107648851	*TAP2*	*Tigger3a/MER96*
32836523-32837522 (1000)	107648851	*TAP2*	*Tigger3a/MER96*
32931739-32933079 (1341)	107648859	3′ncr-*DMB*	*L3/(CT)n/(CA)n*
32931873-32933172 (1300)	107648859	3′ncr-*DMB*	*L3/(CT)n/(CA)n/AluSx*
32935423-32936222 (800)	107648856	*DMB*	*(TCCCAGC)n*
33002673-33005823 (3151)	107648864	*DOA*	*HAL1/MLT10/AluSc/MER5/AluJr*
33005073-33006372 (1300)	107648864	*DOA*	*AluSc/MER5/AluJr/MER5*
33008773-33010672 (1900)	107648863	*DOA*	*MIR/LTR33/MER5*
33010075-33011244 (1170)	107648863	*DOA*	*LTR33/MER5/L2a/L1ME*
33010401-33011244 (844)	107648863	*DOA*	*MER5/L2a/L1ME*
33051181-33057716 (6536)	1105999562	*DOA/DPA1*	*HERVK22-int*
33052612-33056830 (4219)	1105999562	*DOA/DPA1*	*HERVK22-int*
33055396-33057095 (1700)	1105999562	*DOA/DPA1*	*HERVK22-int*
33055689-33056973 (1285)	1105999562	*DOA/DPA1*	*HERVK22-int*

*Jeffreys et al. (2001), Kauppi et al. (2005), Kong et al. (2010), Pratto et al. (2014).*

We searched for *CCTCCCCT* and *ATCCATG* and their reverse complementary sequences *AGGGGAGG* and *CATGGAT*, respectively, to identify their distribution in the centromeric end of MHC class III and the entire class II region from *PRRT1* to *COL11A2* in the human genomic reference sequence GRChr38.p13 (NC_ 000006.12), which is the *DRB1^∗^15:01/DQA1^∗^01:02/DQB1^∗^06:02/DPB1^∗^04:01* haplotype or 7.1AH represented by the MHC-PGF homozygous cell line (Supplementary Table 1) previously described by Horton et al. (2008). We detected 204 copies of the four motifs with 50 to 68.7% of them within different repeat elements (Table 9); the highest percentages were for *CCTCCCCT* within simple repeats (16.1%), *AGGGGAGG* within *MIR* (11.4%), *ATCCATG* within *L1* (43.3%), and *CATGGAT* within *L1* (33.9%). The *MIR* element was also near many of these motifs, as were several different TEs from the TcMar-Tigger, hAT-Charlie ERVL-MaLR, and ERV1 repeat families such as *Charlie*, *Tigger*, *MER20*, *MER5*, and *THE* elements as well as the ancient *LTR16* and *ERVL-E-int* of the ERVL family (Supplementary Table 10).

**TABLE 9:** Number of PRDM9 motifs located in the repeat elements distributed from *RNF5* to *RING1* in the extended MHC II genomic region.

RepName	RepClass	RepFamily	Number of PRDM9 motifs			
			*CCTCCCCT*	*AGGGGAGG*	*ATCCATG*	*CATGGAT*
*Charlie1a*	DNA	hAT-Charlie			1	1
*MamRep38*	DNA	hAT		1		
*MER1*	DNA	hAT-Charlie		1		1
*MER2*	DNA	TcMar-Tigger			2	1
*MER6*	DNA	TcMar-Tigger				1
*MER44*	DNA	TcMar-Tigger			1	2
*Tigger2*	DNA	TcMar-Tigger		1	1	2
*MER91*	DNA	hAT-Tip100	1			
*ORSL*	DNA	hAT-Tip100				1
*L1*	LINE	L1	4	2	29	21
*L2*	LINE	L2	2	2	2	
*L3*	LINE	CR1		1		
*HERVFH19-int*	LTR	ERV1	1			
*HUERS-P2-int*	LTR	ERV1			1	
*LTR12*	LTR	ERV1		1		
*MER4*	LTR	ERV1		1	1	
*MER52*	LTR	ERV1	1			
*MER11*	LTR	ERVK		2		
*HERVK3-int*	LTR	ERVK				1
*MLT2*	LTR	ERVL		1		
*MLT1*	LTR	ERVL-MaLR	2			1
*Alu*	SINE	Alu	1	2	3	1
*MIR*	SINE	MIR	2	5	3	1
*MamSINE1*	SINE	tRNA-RTE				1
Simple_repeat			5	2	2	3
G-rich, GA-rich	Low_complexity					2
Non-repeat region			12	22	21	22
Total number of copies			31	44	67	62

The locations of the PRDM9 recombination motifs are shown in Figure 1 relative to the positions of recombination hotspots, the SNP-density XOs, the *Alu* and *SVA* indels, *LTR*, *MER*, *L1*, and other TE location tags, the centromeric MHC class III genes from *PRRT1* to *BTNL2*, and the HLA class II genes, *MTCO3P1* and *COL11A2* in the MHC class II region. Of the 31 *CCTCCCCT* and 44 *AGGGGAGG* 8mers, 20 and 28 of them, respectively, were in the class II region between the *HLA-DRB1* gene and the *COL11A2* gene. Of these, 17 *CCTCCCCT* and 12 *AGGGGAGG* were in the previously identified recombination hotspots near *MTCO3P1*, within or bordering the *TAP2/PSMB8/TAP1/PSMB9* region, on either side of *HLA-DMB* and *-DMA*, within and flanking *-DOA*, and within the *HLA-DP* gene cluster. Six copies of *CCTCCCCT* and seven copies of *AGGGGAGG* were within the *COL11A2* gene sequence, with only one copy each of the PRDM9 suppressive motifs, *ATCCATG* and *CATGGAT*. Many of these motifs are also found near the SNP-density XO nucleotides.

## Discussion

Haplotype shuffling (SNP-density XOs) at the MHC haplotype boundaries has received relatively little attention (Smith et al., 2006; Traherne et al., 2006; Lam et al., 2013; Larsen et al., 2014; Kulski et al., 2021) when compared with the much greater focus on genotyping SNPs and applying LD statistical analysis to estimate haplotypes (Ahmad et al., 2003; Miretti et al., 2005; Blomhoff et al., 2006; de Bakker et al., 2006; Baschal et al., 2012; Lam et al., 2015). However, several historical studies show that statistically inferred haplotype sequences often miss the importance of CPSs of the CEH (Alper et al., 2006) and AH (Dawkins et al., 1999) in matching donors and recipients for transplantations and for identifying the haplotypes involved in autoimmune diseases (Dawkins et al., 1983) such as T1D (Alper and Larsen, 2017). The present study has taken advantage of the Norman et al. (2017) phased haplotype sequences to examine SNP-density XO points to measure haplotype shuffling in the class II region. The MHC haplotype boundaries or junctions are potential “hotspots” in genome-wide association disease studies. Previously, we investigated the occurrence of TE indels and haplotype exchanges in class I genomic region (Kulski et al., 2021) and now broadened our analysis to TE indels and haplotype switching at the junctions between SNP-rich and SNP-poor blocks in the class II region, covering 620 kb of genomic sequence from the *HLA-DRB1* gene to the *COL11A2* gene (Figure 1). Haplotype shuffling at more than 50 sequence locations was indicated by various genomic markers, including the HLA-class II alleles, *MTCO3P1* alleles, and 42 of 44 TE markers listed in Table 3. The HLA-class II alleles for *HLA-DRB1*, *-DRB2*, *-DRB3*, and -*DRB4* and -*DRB5*, *-DQA1*, *-DQB1*, *-DPA1*, and -*DPB1* were determined by Norman et al. (2017), but to better assess the structure of the haplotype changes, we also included the alleles for *HLA-DQA2*, -*DQB2*, *-DOB*, *-DOA*, *-DPB2*, and *-DPA3* and the pseudogene *MTCO3P1*. In general, the total number of alleles for each of these HLA-class II gene loci are regularly updated and presented by the IPD-IMGT/HLA Database (Robinson et al., 2019) and show that the greatest SNP diversity occurs in the 82-kb genomic region from *HLA-DRB1* (2,838 alleles) to *HLA-DQB1* (1,930 alleles) and to a lesser extent in the 268-kb genomic region from *HLA-DQA2* (38 alleles) to *HLA-DOA* (12 alleles). *HLA-DPB1* at the centromeric end of the MHC class II region has generated 1,654 alleles, whereas the neighboring *HLA-DPA1* gene is less diverse with 216 alleles.

The haplotype estimations and population frequencies of five haplotypic Alu indels, *AluORF10*, *AluDRB1*, *AluDQA1*, *AluDQA2*, and *AluDPB2* (Table 3), were investigated previously in Caucasians, Japanese (Kulski et al., 2010), Chinese Han (Shi et al., 2014), and 12 other Chinese ethnic populations (Cun et al., in preparation). The population frequencies for three of these *Alu* and five others were also reported by The 1000 Genomes Project Consortium et al. (2015a, b) using data from 2,504 unrelated individuals from 26 populations around the world. *AluDQA1* and *AluDRB1* belong to the AluY subgroup, and *AluDQA2*, *AluDPB2*, and *AluORF10* are within the youngest AluYa5 or AluYb8 subgroup (Kulski et al., 2010). Whereas *AluDQA1* appears to be the oldest of the five Alu indels based on its subfamily sequence and for having the highest frequency in different populations and for its association with most of the different *HLA-DR* supertypes (Supplementary Table 5), the frequency of the *AluDQA2* insertion was higher in the Caucasians than in the Chinese or Japanese populations, which supports the hypothesis that it originated in Caucasians (Kulski et al., 2010; Shi et al., 2014). Moreover, five of the 10 *AluDQA2* insertions were linked to four of 17 *AluDQB1* insertions and to five of the 10 8.1 Ancestral haplotypes *HLA-A1-B8-C7-DRB3-DQ2* (Supplementary Table 4), which is a common European haplotype (Aly et al., 2006; Smith et al., 2006; Gambino et al., 2018). The *AluDRB1* indel has a wide frequency range from 0.10 to 0.455 and a strong percentage association with only *HLA-DRB1^∗^15* and -*DRB1^∗^16* in most populations studied so far. These results confirm that the *AluDRB1* insertion probably originated in an ancestral *HLA-DRB1* allele as a progenitor of the *DR51* supertypes (Kulski et al., 2010), which contained *HLA-DRB1^∗^15* or -*DRB1^∗^16* (Andersson, 1998; Suzuki et al., 2018). The *AluDPB2* insertion also has a wide frequency range from 0.278 to 0.574 in 15 populations but with low- to high-level percentage associations with many different *HLA-DRB1* alleles (Supplementary Table 5). This is not surprising because the *AluDPB2* locus is 536 kb from the *HLA-DRB1* locus, with the likelihood of numerous ancient recombination events occurring in between the two loci. In contrast, the *AluORF10* had a strong association with *HLA-DRB1^∗^15*, mostly in Caucasians (89.1%) and a strong association with *HLA-DRB1^∗^16* in eight East Asian populations (Cun et al., in preparation). It is evident from this and previous studies that the closer the dimorphic Alu is to the *HLA-DRB1* locus, the stronger the haplotypic linkage/association, hitchhiking, and recombination resistance (Kulski et al., 2010; 2011). For example, the *AluDRB1* that is most strongly associated with *HLA-DRB1^∗^15* and *HLA-DRB1^∗^16* is located within 14 kb of the *HLA-DRB1* locus, whereas *AluORF10* and *AluDP2*, which are 233 and 536 kb, respectively, from the *DRB1* locus, are associated with many more different *DRB1* alleles. Thus, these five genotyped and haplotyped dimorphic Alu elements are genomic markers that provide evolutionary and “identical by descent” lineage evidence about the common ancestral state, diversity, and genomic rearrangements of the MHC class II region.

The 660-bp *MTCO3P1* pseudogene (NCBI gene ID 404026) was haplotyped as a non-HLA class II allelic marker because of high-frequency haplotype exchanges in the vicinity of its locus between the genomic loci of *HLA-DQB1* and *HLA-DQA2*. *MTCO3P1* has a high sequence identity with cytochrome c oxidase III (NCBI gene ID 4514) in the mitochondrial DNA, and there are numerous other *MTCO3* pseudogene loci distributed throughout the human genome (chromosomes 2, 3, 4, 7, 9, and 16 and Supplementary Figure 2). We identified 10 *MTCO3P1* sequence variants (Supplementary Table 3) in 84 sequences, with gaps or insufficient sequence information available in the remaining 11 sequences. *MTCO3P1^∗^06*, *MTCO3P1^∗^08*, and *MTCO3P1^∗^10* appear to be haplospecific, whereas the other seven variants are haplotypic with *MTCO3P1^∗^03* linked to nine different *HLA-DRB1* haplotypes (Table 2) and to the 7-kb #44 indel composed of at least 10 other TE family members (Table 4 and Supplementary Table 4). Most of the haplotype shuffling was detected in the genomic region between *MTCO3P1* and *HLA-DQB3* in 31 paired sequence comparisons and between the *HLA-DQB1* and *MTCO3P1* loci in 11 cases (Figure 1 and Supplementary Table 9). Although we identified 10 alleles for *MTCO3P1*, there are at least 102 sequence variants archived at the National Institute on Aging Genetics of Alzheimer’s Disease Data Storage Site—Genomics database^12^ that possibly could be linked to many more MHC class II haplotypes. The *MTCO3P1* genomic sequence might be involved haplotypically with Alzheimer’s disease and Alzheimer’s disease-related neuropathologies (National Institute on Aging Genetics of Alzheimer’s Disease Data Storage Site—Genomics database). In this regard, the GWAS survey by Chesmore et al. (2018) revealed that *MTCO3P1* was the third most pleiotropic sequence in the human genome with 32 phenotype associations after the top-ranking *ABO* gene on chromosome 9 with 39 phenotype associations—a gene that forms the basis of the ABO blood group diversity. The *HLA-DRB1*, *-DQB1*, and -*DQA1* genes also were among the 10 top-ranking pleiotropic genes in the Chesmore et al. (2018) study and, together with the *MTCO3P1* variants, might be associated with systemic lupus erythematosus, T1D, immunoglobulin A nephropathy, Crohn’s disease, multiple sclerosis, narcolepsy, and systemic sclerosis among various other disease phenotypes. Some of the *MTCO3P1* alleles together with dimorphic haplotypic TE markers such as the *LTR13-DQB1*, *AluMT2*, *AluMT3*, and the 7-kb #44 indel might be useful genomic markers for subdividing MHC class II haplotype disease associations into different categories, such as the *HLA-DRB1^∗^03/DQA1^∗^03/DQB1^∗^03* haplotypes associated with TID and/or autoimmune Addison’s disease (Table 2, Pani et al., 2002; Gambelunghe et al., 2005). The *LTR13-DQB1* indel was linked to six different *DRB1-MTCO3P1* haplotypes (Supplementary Table 4), some of which might be useful for predicting the onset of T1D and/or Addison’s disease in multiple populations (Valdes et al., 2012; Vadva et al., 2019). In addition, there are four *TcMar-Tigger* DNA elements (Smit and Riggs, 1996) located between *MTCO3P1* and *HLA-DQB3*, including a 2,411-bp *Tigger1* sequence adjoining the *HLA-DQB3* pseudogene locus in all of the haplotypes examined in this study. *Tigger* repeat sequences can generate microRNAs for the regulation of gene expression at the post-translational level (Qin et al., 2015), and they have been found overrepresented in cell-free DNA extruded from cultured human bone osteosarcoma cells (Bronkhorst et al., 2018).

The identification of dimorphic TEs near the junctions of duplicated genes (Kulski et al., 2021) and at ectopic and meiotic recombination sites (Myers et al., 2010; Altemose et al., 2017; Kent et al., 2017) as well as at SNP-density XO sites (Figure 1) further emphasizes their role in contributing to genomic diversity. The two highest SNP-density levels between different MHC class II haplotypes were seen in the genomic region between *HLA-DRB1* and *HLA-DOB* (Figure 3), which also contained 13 of the 15 *Alu* indels, two of the five *SVA* indels, two *MER11* indels, two *LTR5* indels, and a single indel location each for *LTR13*, *LTR33*, and *LTR42* (Figure 1 and Table 3) and 44 of the 98 SNP-density XO points (Supplementary Table 9) in the MHC class II region. This connection between TE indels and SNP-density XOs confirms our previous finding that the dimorphic *Alu* and *SVA* are located close to or within putative recombination hotspots throughout the MHC classes I, II, and III genomic regions (Kulski et al., 2011, 2021), suggesting that they might be involved in DNA repair in response to genomic stress and damage (Kulski et al., 2000a). Although the expression of most *Alu* elements in the human genome is silenced by methylation, they are transcriptionally active in germ cells during early development and in response to cellular and genomic stress and damage as a result of heat shock (Schmid, 1996), viral infections (Chu et al., 1998; Tucker and Glaunsinger, 2017), autoimmune diseases (Yüksel et al., 2016; Wu et al., 2019), and cancer pathogenesis (Moolhuijzen et al., 2010; Kaczkowski et al., 2016). Many *Alu* elements of the *AluJ*, *AluS*, and *AluY* subfamilies are transcriptionally active with highly expressed self-cleaving ribozyme activity during T-cell activation and thermal and endoplasmic reticulum stress (Hernandez et al., 2020). Furthermore, Wang et al. (2017) identified three TE indels, *Alu-5072*, *Alu-5075*, and *SVA-282*, in the class II region as potential enhancers for *HLA-DRB5*, *HLA-DQB1-AS1*, and *HLA-DPB2* associated with GWAS phenotypes of lymphoma, Hodgkin lymphoma, and chronic hepatitis B infection, respectively. *Alu-5057* is probably the *AluDRB1* indel at the 5′ end of *HLA-DRB1*, whereas *SVA-282* is likely the *SVA-DPA1* indel at the 3′ end of *HLA-DPA1* (Figure 1). Thus, the question remains whether the other 19 Alu and six SVA indels identified in the extended class II region in this study (Table 3) also have enhancer functions as reported by Wang et al. (2017) for *Alu-5072*, *Alu-5075*, and *SVA-282*. On the basis of these findings, the transcriptional activity and role of *Alu* and *SVA* in the human MHC during epigenetic regulation need to be investigated further and better defined.

There are ∼121 LTR sequences interspersed between the *DRB1* and *COL11A2* gene loci, but few have been investigated specifically as genetic risk markers in disease association studies. *DQ-LTR13* was associated with *DRB1^∗^0401* T1D susceptibility (Bieda et al., 2002; Krach et al., 2003) and autoimmune Addison’s disease due to an LD with *DQB1^∗^0302* and *DRB1^∗^0403* (Pani et al., 2002; Gambelunghe et al., 2005). HERV LTRs found in the class II region such as *MER11*, *MER41*, *MER44*, *LTR5*, *LTR9*, and *LTR12* are known to regulate the transcription of neighboring genes outside the MHC genomic region (Bi et al., 1997; Bièche et al., 2003; Buzdin et al., 2006; Chuong et al., 2017; Daskalakis et al., 2018). The solitary 482-bp *LTR42* indel that is located 1-kb telomeric of the 3′ end of *HLA-DOB* coding gene is positively linked to the *AluDOB2* insertion and negatively linked to the *AluDOB1* insertion (Table 5) and might act to regulate the transcriptional and/or translational activity of either *HLA-DQB2* or *HLA-DOB*. There are only two HERVs in the class II region centromeric of *HLA-DRB1* that are greater than 5-kb in length; the *LTR12* (526 bp)/*HERVK22* (6,805 bp) and *LTR9* (666 bp)/*HUERS-P3-int* (5589 bp)/*LTR9* (666 bp) sequences. The *HERVK22* sequence is located near the *SVA-DPA1* indel at the 3’ end of the *HLA-DPA1* gene, and it is a putative ancestral meiotic recombination hotspot with SNP-density XOs (haplotype shuffling) occurring within its sequence (Supplementary Table 9). The *HUERS* sequence is fragmented and interrupted by a 1-kb *LTR5* insertion in all of the 90 haplotype sequences that were examined, and it is located within the *HLA-DPB2* pseudogene and less than 1 kb from the *AluDPB2* indel (Supplementary Figure 1). There are also HERVs (*HERVK3*, *HERV9*, *ERVLE*, *HERVIP10*, and *HERVK14*) located in the *HLA-DR* super haplotype region (Supplementary Table 7, Andersson et al., 1998; Doxiadis et al., 2008; Horton et al., 2008) and in the class I region (Kulski et al., 1999a, 2021). These and other ERVs provide promoter and enhancer exaptation and non-coding transcripts of viral accessory proteins as regulatory units (Buzdin et al., 2006, 2017; Daskalakis et al., 2018) that might have a pathogenic role in several autoimmune diseases such as rheumatoid arthritis and systemic lupus erythematosus by providing epitopes, superantigens, and/or hypomethylation motifs (Moyes et al., 2007; Balada et al., 2010; Tugnet et al., 2013; Trela et al., 2016). The diseases associated with MHC haplotypes (Dawkins et al., 1983; Lokki and Paakkanen, 2019) are still poorly defined, and current knowledge about genomic disease associations remains largely at the level of SNPs and alleles in GWAS. In this regard, structurally polymorphic TEs are potential haplotype disease markers to associate particular MHC haplotypes and disease traits.

In previous studies, the major recombination hotspots in the MHC class II region were identified between *HLA-DQB1* and *HLA-DQA2* (near *MTCO3P1*); within intron 2 of *TAP2*; between *TAP2* and *HLA-DMB*; between *BRD2* and *HLA-DOA*; between *HLA-DOA* and *HLA-DPA1*; and between *HLA-DPB1* and *RING1* (Jeffreys et al., 2001; Cullen et al., 2002; Miretti et al., 2005; Table 8). Our comparative sequence analysis of 121 different haplotype pairs revealed 98 unique XO sites between SNP-poor and SNP-rich genomic segments with considerable haplotype shuffling in the proximity of the putative recombination hotspots (Figure 1). The majority of SNP-density XO sites occurred across various regions, including within *HLA-DRB1*; between the *HLA-DRB1* and *HLA-DQA1* loci; between *HLA-DQA1* and *HLA-DQB1*; in the vicinity of *MTCO3P1* between *HLA-DQB1* and *HLA-DQB3;* between *HLA-DQB2* and *HLA-DOB*; between *DOB* and *TAP2*; within *TAP2*; and between *HLA-DOA* and *HLA-DPA1* where eight XO sites were found within a *HERVK22* sequence. The SNP XO sites were located mostly outside of TEs, but 43 of the 98 unique XO-SNPs were found within TE sequences, including within different Alu subfamilies, L1 subfamilies, *MER20*, *LTR1*, *LTR5*, *LTR19*, *LTR33*, *LTR41*, *Tigger3b*, *MLT1H*, *MER3*, *SVA-DPA1*, and *HERVK22*-int (Supplementary Table 9). We also determined the genomic positions of the PRDM9-recombination suppression sequence motif *ATCCATG/CATGGAT* and the PRDM9 recombination activation partial binding motif *CCTCCCCT/AGGGGAG* in the class II region of the human reference genome (NC_ 000006) relative to published meiotic recombination positions (Figure 1).

A possible limitation in our comparative analysis between the SNP XO sites and recombination sites (Figure 1) is that there are large differences between our data and those of others, such as with methodologies, amounts of data analyzed, and the genotypes or haplotypes studied. However, our comparative analysis generally supports and extends the previous observations of the centromeric class II region by Larsen et al. (2014) for 158 common European haplotypes, and we concur with them that the SNP density XOs or haplotype break point locations and frequencies vary significantly between different CEH/AHs. Also, the exact historical sequence recombination leading to the breakage or shuffling of a dominant haplotype based solely on SNP transitions between high and low (or absent) densities is difficult to localize precisely or with confidence. We did not have sufficient sequences with the same haplotypes to demonstrate that some dominant sequences break down gradually in many locations, whereas some others break down at specific locations (Larsen et al., 2014). Instead, we found that SNP-density XOs were mostly at specific locations, as shown in Figure 3 and listed in Supplementary Table 9. Most of the XO sites that we observed depended on which haplotypes were compared, and the differences between various haplotypes are probably due to different CEH/AH expansion timelines and recombination events. Taken together, our study and that of Larsen et al. (2014) show substantial haplotype shuffling between different polymorphic blocks and suggest the presence of numerous putative ancestral recombination sites across the class II region located between various HLA class II genes.

Of 125 haplotype-pairwise-sequence comparisons in this study, 121 showed evidence of haplotype shuffling at least once within the 620-kb genomic region between the telomeric *HLA-DRB1* and the centromeric *COL11A2* gene (Supplementary Table 9). Much of this exchange occurred between or in close vicinity to the PRDM9-mediated recombination activation motif *CCTCCCCT/AGGGGAG* and/or recombination suppression motif *ATCCATG/CATGGAT* (Figure 1) that is recognized by the PRDM9-mediated recombination machinery to alter chromatin structure and PRDM9 initiated meiotic recombination (Myers et al., 2010; Altemose et al., 2017; Parvanov et al., 2017). Our analysis of the PRDM9 motifs was limited mainly to the reference genome GRChr38.p13 for this study, but we found some site differences between MHC haplotypes that still need to be evaluated in greater detail (data not shown). Both the recombination and anti-recombination motifs are widely distributed throughout the class I and class II genomic regions (Kulski et al., 2021), with 50% or more found within repeat elements. The anti-recombination motifs mostly involved the L1 fragmented repeats, whereas the recombination activation partial binding motifs were distributed more widely between L1, MIR, and simple repeats (Table 9). In addition, many have accumulated within regions of previously identified ancestral meiotic recombination sites (Table 8) and in proximity to regions of pseudogene conversions and near the loci of the dimorphic *Alu* and *SVA* insertions. Multiple recombinations in the MHC might be relatively rare events (Cullen et al., 1997, 2002; Smith et al., 2006), but we found multiple SNP-density XOs within the class II region in 40 of 121 (33%) pairwise sequence analyses of different haplotypes (Table 7) including zero to three XOs in the comparison between haplotype ID_51 and three other haplotypes (ID_41, _62, and _68) in Figure 3. The present study on haplotype shuffling and TE indels within the class II region extends our previous study within the class I region using the same Norman et al. (2017) haplotype sequences. The important class III region that harbors many immune regulatory genes was not examined in detail here, although we found evidence of haplotype exchanges in the genomic regions between *MICB* and *TNF*, *C4/C2* duplication region (data not shown), and between *NOTCH4* and *TSBP1* (Figure 1), similar to the SNP-density XO regions described by Lam et al. (2013) in the proximity of the meiotic hotspots reported by Cullen et al. (1997, 2002).

Comparative genomic analysis of haplotype diversity and shuffling advances our understanding of the evolutionary history of human genomic architecture and the variability of population structures and ancestry. Our previous analysis of haplotype shuffling in the MHC class I region (Kulski et al., 2021) and the class II region in this study further highlights the metameric design of the MHC genomic region characterized by the presence of duplicated HLA-genes, *LTR16/HERV16* duplications, and various repeating TEs embedded within distinct subgenomic segments (Kulski et al., 1999b, 2000a,b, 2002, 2004, 2021), polymorphic frozen blocks (Dawkins et al., 1999), and conserved regions of framework genes (Amadou, 1999). These segments and blocks have evolved with considerable haplotype shuffling of CPSs by meiotic recombination, unequal recombinations, and gene conversions often involving TEs. In this context, it appears that the location of many of the dimorphic TEs within the MHC is the remains of DNA repair “sutures” or “scars” in response to genomic stress and damage and/or meiotic recombinations and unequal XO events (Schmid, 1996; Kulski et al., 2000a; Hernandez et al., 2020). The length of class II genomic region from *HLA-DRA1* to -*DPB2* (∼689 kb) is ∼3 × shorter than the class I region from *HLA-F* to *MICB* (∼1.8 Mb), but it seems to have a greater frequency of multiple haplotype shuffling events at the junctions between SNP-poor and SNP-rich blocks (Kulski et al., 2021, and this study). This may be due in part to differences in CPSs, TEs, genomic structure, and evolutionary age of class I and class II regions; the primate class II allelic lineages appear to be much older than those of the class I allelic lineages (Andersson, 1998; Kulski et al., 2000b, 2004; Tìšickı and Vinkler, 2015). Also, the mean recombination rate is ∼4 × greater in the class II region than in the class I region (Radwan et al., 2020). The information gathered so far for putative ancestral recombination sites and haplotype shuffling between different polymorphic blocks in this and previous studies is still largely based on the availability of a limited number of MHC genomic sequences representing approximately 50–80 MHC ancestral haplotypes, a very small fraction of the thousands of different haplotypes that are distributed in worldwide populations (Goodin et al., 2018). Many more MHC genomic haplotype sequences need to be added to those already generated by Horton et al. (2008), Lam et al. (2015), and Norman et al. (2017) for better informed comparative genomics, population studies, disease associations, and genetic manipulations such as knock-in and knock-out in regulatory and functional studies. The advancement of new sequencing strategies (Cheng et al., 2021; Ebert et al., 2021; Human Genome Structural Variation Consortium et al., 2021) might allow these types of analyses to be extended to many more fully phased HLA haplotypes in the future. Such work will lead to a better understanding of the role of TEs and the different DNA processes involved in the evolution of the human MHC haplotypes, IBD segments, and their association with autoimmune disorders and other disease traits.

## Data Availability Statement

The original contributions presented in the study are included in the article/Supplementary Material, further inquiries can be directed to the corresponding author/s.

## Author Contributions

JK carried out the analyses of the repeat elements, haplotype sequence comparisons, SNP-density XOs, and interpretation of the data and wrote the manuscript. SS and TS analyzed and interpreted parts of the data and provided the alleles for the classical and non-classical HLA class II genes, HLA pseudogenes, and the *MTCO3P1* pseudogene and provided the SNP density plots. All authors checked the final version of the manuscript.

## Conflict of Interest

The authors declare that the research was conducted in the absence of any commercial or financial relationships that could be construed as a potential conflict of interest.

## Footnotes

1 https://www.ncbi.nlm.nih.gov/bioproject/

2 https://www.ncbi.nlm.nih.gov/assembly/GCF_000001405.39/

3 http://asia.ensembl.org/Homo_sapiens/Info/Index

4 https://genome.ucsc.edu/cgi-bin/hgGateway

5 http://www.repeatmasker.org/cgi-bin/WEBRepeatMasker

6 https://www.ebi.ac.uk/ipd/imgt/hla/

7 https://www.ncbi.nlm.nih.gov/genbank/

8 http://www.genecodes.com

9 http://pipmaker.bx.psu.edu/cgi-bin/multipipmaker

10 https://www.ebi.ac.uk/Tools/msa/tcoffee/

11 https://www.ncbi.nlm.nih.gov

12 https://beta.niagads.org/genomics/app/record/gene/ENSG00000235040

## Abbreviations

AH: ancestral haplotype
CEH: conserved extended haplotype
CPS: conserved polymorphic sequence
CR1: chicken repeat 1
EMBL-EBI: European Molecular Biology Laboratory - The European Bioinformatics Institute
ERV: endogenous retrovirus
GWAS: genome-wide association study
HERVs: human endogenous retrovirus
HLA: Human Leukocyte Antigen
IBD: identical by descent
indel: insertion-deletion
LD: linkage disequilibrium
LINES: long interspersed retrotransposable elements
LTR: long terminal repeat
MER: medium reiterated repeat
MHC: major histocompatibility complex
MIR: mammalian-wide interspersed repeat
NCBI: National Center for Biotechnology Information
Rec Site: recombination site
SINES: short interspersed retrotransposable elements
SNP: single nucleotide polymorphism
STR: short tandem repeat
SVA: short interspersed nuclear element–VNTR–Alu
T1D: type 1 diabetes
TE: transposable element
UCSC, University of California: Santa Cruz
XO: crossover.
