# Supplementary material for: Haplotype Shuffling and Dimorphic Transposable Elements in the Human Extended Major Histocompatibility Complex Class II Region
Source: Front Genet. 2021 May 28;12:665899. doi: 10.3389/fgene.2021.665899 (PMC8193847; doi:10.3389/fgene.2021.665899)
Supplement: Supplementary file 3 [file Table_3.DOCX]

**Supplementary Table 3: Ten *MTCO3P1* allelic sequences (660 bp) extracted from MHC haplotype sequences provided by Norman et al., (2017).**

>MTCO3P1*01

ATGACCCACCAAACACTTGCCTACCATATAGCTGTACCTAGCCCTTGACCACTAACAGGAGCTCTCTCGGCTCTCCTAATAACATCTGGCCTGGCCATATGATTTCACTTTAATTCTACCACTCTTTTAACTTTAGGCCTACTAACCAACACACTGACTATACATCAGTGATGACGTGATATTGTCCGAGAAAGTATATTCCAAGGCCACCACACAACAATTGTCCTAAAAGACCTCCGATACGGGATGCTCCTATTTATTACCTCAGAAGTATTCTTCTTCGCTGGTTTTTTTCTGGGCATTCTATCACTCCAGTTTAGCACCGACCCCAGAATTAGGAGGACATTGACCCCCAACAGGTATTCTTCCCCTGAACCCTTTAGAAGTATCTCTCCTAAATACGTCTGTATTACTTGCATCAGGAGTTTCAATTACTTGAGCCCATCACAGCCTAATAGAAGGTAACCAAAAACAAATAATTCAAGCAGTACTTACCACGATCCTCTTAGGAGTCTATTTCACTCTTCTACAAGTCTCAGAATATTTCGAGGCCCCTTTATTATCTCTGATGGAATCTATGGCTCAACATTCTTTGTAGCCACAGGCTTTCATGGACTTCATGTTATTATTGGATCAACAATTCTCACCATCTGCCTTCTT

>MTCO3P1*02_NC_000006.12

ATGACCCACCAAACACTTGCCTACCATATAGCTGTACCTAGCCCTTGACCACTAACAGGAGCTCTCTCGGCTCTCCTAATAACATCTGGCCTGGCCATATGATTTCACTTTAATTCTACCACTCTTTTAACTTTAGGCCTACTAACCAACACACTGACTATACATCAGTGATGACGTGATATTGTCCGAGAAAGTATATTCCAAGGCCACCACACAACAATTGTCCTAAAAGACCTCCGATACGGGATGCTCCTATTTATTACCTCAGAAGTATTCTTCTTCACTGGTTTTTTTCTGGGCATTCTATCACTCCAGTTTAGCACCGACCCCAGAATTAGGAGGACATTGACCCCCAACAGGTATTCTTCCCCTGAACCCTTTAGAAGTATCTCTCCTAAATACGTCTGTATTACTTGCATCAGGAGTTTCAATTACTTGAGCCCATCACAGCCTAATAGAAGGTAACCAAAAACAAATAATTCAAGCAGTACTTACCACGATCCTCTTAGGAGTCTATTTCATTCTTCTACAAGTCTCAGAATATTTCGAGGCCGCTTTATTATCTCTGATGGAATCTATGGCTCAACATTCTTTGTAGCCACAGGCTTTCATGGACTTCATGTTATTATTGGATCAACAATTCTCACCATCTGCCTTCTT

>MTCO3P1*03

ATGACCCGCCAAACTCATGCCTACCATATAGCTGTACCTAGCCCTTGACCACTAACAGGAGCTCTCTCGGCTCTCCTAATAACATCTGGCCTGGCCATATGATTTCACTTTAATTCTACCACTCTTTTAACTTTAGGCCTACTAACCAACACACTGACTATACATCAGTGATGACGTGATGTTGTCCGAGAAAGTGTATTACAAGGCCACCACACAACAATTGTCCTAAAAGACCTCTGATACGGGATGCTTCTATTTATTATCTCAAAAGTATTCTTCTTCGCTGGTTTTTTTCTGGGCATTTTATCACTCCAGTTTAGCACCGACCCCAGAATTAGGAGGACATTGACCCCCAACAGGTATTCTTCCCCTGAACCCTTTAGAAGTATCTCTCCTAAATACGTCTGTATTACTTGCATCAGGAGTTTCAATTACTTGAGCCCACCACAGCCTAATAGAAGGTAACCAAAAACAAATAATTCAAGCAGTACTTATCACGATCCTCTTAGGAGTCTATTTCACTCTTCTACAAGTCTCAGAATATTTCGAGGCCCCCTTTATTATCTCTGATGGAATCTATGGCTCAACATTCTTTGTAGCCACAGGCTTTCATGGACTTCATGTTATTATTGGATCAACAATTCTCACCATCTGCCTTCTT

>MTCO3P1*04

ATGACCCGCCAAACTCATGCCTGCCATATAGCTGTACCTAGCCCTTGACCACTAACAGGAGCTCTCTCGGCTCTCCTAATAACATCTGGCCTGGCCATATGATTTCACTTTAATTCTACCACTCTTTTAACTTTAGGCGTGCTAACCAACACACTGACTATACATCAGTGATGACGTGATATTGTCCGAGAAAGTATATTCCAAGGCCACCACACAACAATTGTCCTAAAAGACCTCTGATACGGGATGCTCCTATTTATTATCTCAGAAGTATTCTTCTTCGCCGGTTTTTTCTGGGCATTCTATCACTCCAGTTTAGCACCGACCCCAGAATTAGGAAGACATTGACCCCCAACAGGTATTCTTCCCCTGAACCCTTTAGAAGTATCTCTCCTAAATACGTCTGTATTACTTGCATCAGGAGTTTCAATTACTTGAGCCCATCACAGCCTAATAGAAGGTAACCAAAAACAAATAATTCAAGCAGTACTTACCACGATCCTCTTAGGAGTCTATTTCACTCTTCTACAAGTCTCAGAATATTTCGAGGCCCCTTTATTATCTCTGATGGAATCTATGGCTCAACATTCTTTGTAGCCACAGGCTTTCATGGACTTCATGTTATTATTGGATCAACAATTCTCACCATCTGCCTTCTT

>MTCO3P1*05

ATGACCCACCAAACACTTGCCTACCATATAGCTGTACCTAGCCCTTGACCACTAACAGGAGCTCTCTCGGCTCTCCTAATAACATCTGGCCTGGCCATATGATTTCACTTTAATTCTACCACTCTTTTAACTTTAGGCCTACTAACCAACACACTGACTATACATCAGTGATGACGTGATATTGTCCGAGAAAGTATATTCCAAGGCCACCACACAACAATTGTCCTAAAAGACCTCCGATACGGGATGCTCCTATTTATTACCTCAGAAGTATTCTTCTTCACTGGTTTTTTTCTGGGCATTCTATCACTCCAGTTTAGCACCGACCCCAGAATTAGGAGGACATTGACCCCCAACAGGTATTCTTCCCCTGAACCCTTTAGAAGTATCTCTCCTAAATACGTCTGTATTACTTGCATCAGGAGTTTCAATTACTTGAGCCCATCACAGCCTAATAGAAGGTAACCAAAAACAAATAATTCAAGCAGTACTTACCACGATCCTCTTAGGAGTCTATTTCACTCTTCTACAAGTCTCAGAATATTTCGAGGCCCCTTTATTATCTCTGATGGAATCTATGGCTCAACATTCTTTGTAGCCACAGGCTTTCATGGACTTCATGTTATTATTGGATCAACAATTCTCACCATCTGCCTTCTT

>MTCO3P1*06

ATGACCCACCAAACACTTGCCTACCATATAGCTGTACCTAGCCCTTGACCACTAACAGGAGCTCTCTCGGCTCTCCTAATAACATCTGGCCTGGCCATATGATTTCACTTTAATTCTACCACTCTTTTAACTTTAGGCCTACTAACCAACACACTGACTATACATCAGTGATGACGTGATATTGTCCGAGAAAGTATATTCCAAGGCCACCACACAACAATTGTCCTAAAAGACCTCCGATACGGGATGCTCCTATTTATTACCTCAGAAGTATTCTTCTTCGCTGGTTTTTTTCTGGGCATTCTATCACTCCAGTTTAGCACCGACCCCAGAATTAGGAGGACATTGACCCCCAACAGGTATTCTTCCCCTGAACCCTTTAGAAGTATCTCTCCTAAATACGTCTGTATTACTTGCATCAGGAGTTTCAATTACTTGAGCCCATCACAGCCTAATAGAAGGTAACCAAAAACAAATAATTCAAGCAGTACTTACCACGATCCTCTTAGGAGTCTGTTTCACTCTTCTACAAGTCTCAGAATATTTCGAGGCCCCTTTATTATCTCTGATGGAATCTATGGCTCAACATTCTTTGTAGCCACAGGCTTTCATGGACTTCATGTTATTATTGGATCAACAATTCTCACCATCTGCCTTCTT

>MTCO3P1*07

ATGACCCACCAAACACTTGCCTACCATATAGCTGTACCTAGCCCTTGACCACTAACAGGAGCTCTCTCGGCTCTCCTAATAACATCTGGCCTGGCCATATGATTTCACTTTAATTCTACCACTCTTTTAACTTTAGGCCTACTAACCAACACACTGACTATACATCAGTGATGACGTGATATTGTCCGAGAAAGTATATTCCAAGGCCACCACACAACAATTGTCCTAAAAGACCTCTGATACGGGATGCTCCTATTTATTATCTCAGAAGTATTCTTCTTCGCTGGTTTTTTCTGGGCATTTTATCACTCCAGTTTAGCACCGACCCCAGAATTAGGAGGACATTGACCCCCAACAGGTATTCTTCCCCTGAACCCTTTAGAAGCACCTCTCCTAAATACATCTGTATTACTAGCATCACGAGTTTCAATTACTTGAGCCCATCACAGTCTAATAGAAGGTAACCAAAAACAAATAATTCAAGCAGTACTTACCACGATCCTCTTAGGAGTCTATTTCACTCTTCTACAAGTCTCAGAATATTTCGAGGCCCCCTTTATTATCTCTGATGGAATCTATGGCTCAACATTCTTTATAGCCACAGGCTTTCATGGACTTCATGTTATTATTGGATCAACAATTCTCACCATCTGCCTTCTC

>MTCO3P1*08

ATGACCCGCCAAACTCATGCCTACCATATAGCTGTACCTAGCCCTTGACCACTAACAGGAGCTCTCTCGGCTCTCCTAATAACATCTGGCCTGGCCATATGATTTCACTTTAATTCTACCACTCTTTTAACTTTAGGCCTACTAACCAACACACTGACTATACATCAGTGATGACGTGATATTGCCCGAGAAAGTATATTCCAAGGCCACCACACAACAATTGTCCTAAAAGACCTCCGATACGGGATGCTTCTATTTATTACCTCAGAAGTATTCTTCTTCGCTGGTTTTTTCCTGGGCATTCTATCACTCCAGTTTAGCACCGACCCAAGAATTAGGAGGACATTGACCCCCAACAGGTATTCTTCCCCTGAACCCTTTAGAAGTATCTCTCCTAAATACGTCTGTATTACTTGCATCACGAGTTTCAATTACTTGAGCCCATCACAGTCTAATAGAAGGTAACCAAAAACAAATAATTCAAGCAGTACTTACCATGATCCTCTTAGGAGTCTATTTCACCCTTCTACAAGT:TCAGAATATTTCGAGGCCCCCTTTATTATCTCTGATGGAATCTATGGCTCAACATTCTTTATAGCCACAGGCTTTCATGGACTTCATGTTATTATTGGATCAACAATTCTCACCACCTGCCTTCTC

>MTCO3P1*09

ATGACCCGCCAAACTCATGCCTACCATATAGCTGTACCTAGCCCTTGACCAGTAACAGGAGCTCTCTCGGCTCTCCTAATAACATCTGGCCTGGCCATATGATTTCACTTTAATTCTACCACTCTTTTAACTTTAGGCCTACTAACCAACACACTGACTATACATCAGTGATGACGTGATATTGCCCGAGAAAGTATATTCCAAGGCCACCACACAACAATTGTCCTAAAAGACCTCCGATACGGGATGCTTCTATTTATTACCTCAGAAGTATTCTTCTTCGCTGGTTTTTTCCTGGGCATTCTATCACTCCAGTTTAGCACCGACCTGAGAATTAGGAGGACATTGACCCCCAGCAGGTATTCTTCCCCTGAACCCTTTAGAAGTATCTCTCCTAAATACGTCTGTATTACTTGCATCACGAGTTTCAATTACTTGAGCCCATCACAGTCTAATAGAAGGTAACCAAAAACAAATAATTCAAGCAGTACTTACCATGATCCTCTTAGGAGTCTATTTCACCCTTCTACAAGTTCAGAATATTTCGATTCCCCCTTTATTATCTCTGATGGAATCTATGGCTCAACATTCTTTATAGCCACAGGCTTTCATGGATTTCATGTTATTATTGGATCAACAATTCTCACCATCTGCCTTCTC

>MTCO3P1*10

ATGACCCGCCAAACTCATGCCTACCATATAGCTGTACCTAGCCCTTGACCACTAACAGGAGCTCTCTCGGCTCTCCTAATAACATCTGGCCTGGCCATATGATTTCACTTTAATTCTACCACTCTTTTAACTTTAGGCCTACTAACCAACACACTGACTATACATCAGTGATGACGTGATGTTGTCCGAGAAAGTGTATTACAAGGCCACCACACAACAATTGTCCTAAAAGACCTCTGATACGGGATGCTTCTATTTATTATCTCAAAAGTATTCTTCTTCGCTGGTTTTTTTCTGGGCATTTTATCACTCCAGTTTAGCACCGACCCCAGAATTAGGAGGACATTGACCCCCAACAGGTATTCTTCCCCTGAACCCTTTAGAAGTATCTCTCCTAAATACGTCTGTATTACTTGCATCACGAGTTTCAATTACTTGAGCCCATCACAGTCTAATAGAAGGTAACCAAAAACAAATAATTCAAGCAGTACTTACCATGATCCTCTTAGGAGTCTATTTCACCCTTCTACAAGT:TCAGAATATTTCGAGGCCCCCTTTATTATCTCTGATGGAATCTATGGCTCAACATTCTTTATAGCCACAGGCTTTCATGGACTTCATGTTATTATTGGATCAACAATTCTCACCACCTGCCTTCTC

The linkage between *MTCO3P1* alleles and HLA-class II gene alleles are shown in **Supplementary Table 2.**
