# Supplementary material for: Haplotype Shuffling and Dimorphic Transposable Elements in the Human Extended Major Histocompatibility Complex Class II Region
Source: Front Genet. 2021 May 28;12:665899. doi: 10.3389/fgene.2021.665899 (PMC8193847; doi:10.3389/fgene.2021.665899)
Supplement: Supplementary file 8 [file Table_8.DOCX]

**Supplementary Table 8. TE markers in the DRB haplotype region**

DRB8:Hap0

DRB9/HERVK3/LTR3

LTR12/HLA-DRB1*08

AluDRB1

AluDQA1a

ORSL/HLA-DQA1

DRB3: [DRB2(6)] COX_Hap2 cell line

DRB9/HERVK3/LTR3

MER52/MSTB

LTR12/HLA-DRB3

MER52/LTR5

LTR12/HERV9

[HLA-DRB2]

HERVK3/LTR3

LTR12/HLA-DRB1*3

DRB2: [DRB3] COX_Hap2 cell line

DRB9/HERVK3/LTR3

LTR12

MER52/Lor1/MER52

LTR5

LTR12/HERV9/LTR12F

[HLA-DRB2]

HERVK3/LTR3

LTR12F/[HLA-DRB1*03]

[HLA-DQA1]

LTR16A/SVA_f-DQB1

[HLA-DQB1]

Zaphod

(L1PA10)

MER70/MER54/ERVLE/MLT1A0

DRB5: [DRB6]

DRB9/HERVK3/LTR3

LTR60/LTR43/MER50

Lor1/MER51/Lor1

HERV9..LTR12F

LTR9/HERV9/LTR12 [HERVIP10]

LTR14

HLA-DRB5

SVAF-DRB5

HERV9/LTR12

LTR12c

[HLA-DRB6]

LTR3/HERVK3/LTR3

LTR12/[HLA-DRB1*15/16]

SVAf-DRB1

AluDRB1

DRB4: [DRB8/DRB7] MANN-Hap4, MCF_HAP5, SSTO_HAP7 cell lines

DRB9/HERVK3/LTR3

LTR60/LTR43/MER50

Lor1

HERV9..LTR12F

MER52/Lor1/MER51/HERV9/LTR12

HLA-DRB4

LTR43 …Lor1

HERV9…LTR12F

Lor1/MER51/Lor1

MER77

LTR14..HERVK14/LTR14

LTR22

MER52..HERV9-int

[DRB1*04/07/09]

DRB7: [DRB4, DRB8] (cellline dbb_hap3, mann_hap4, ssto_hap7)

DRB9/HERVK3/LTR3

[HLA-DRB7]

LTR14..HERVK14/LTR14

LTR22

LTR60/LTR43

Lor1/MER51/

HERV9-int/SVAd-DRB7/HERVK9

[HLA-DRB1*04/07/09]

[HLA-DQA1]

SVA_F-DQA1

DRB8: [DRB4, DRB7] (cell lines mann_hap4, ssto_hap7)

DRB9/HERVK3/LTR3

LTR60/LTR43/MER50

Lor1

HERV9..LTR12F

MER52/Lor1/MER51

HERV9/LTR12

[HLA-DRB4]

MER52/THE1D/

LTR43

Lor1

HERV9…LTR12F

[HLA-DRB8]

Lor1/MER51/

MER77

[HLA-DRB7]

LTR14..HERVK14/LTR14

(L1PA6)

LTR22

LTR60/LTR43

Lor1/MER51/Lor1

HERV9-int/SVAd-DRB7/

[DRB1*04/07/09]

[HLA-DQA1]

SVA_F-DQA1

LTR16A

SVA_e-DQB1

References: Horton et al., (2008); Norman et al., (2017).
