# Supplementary figures and images for: Haplotype Shuffling and Dimorphic Transposable Elements in the Human Extended Major Histocompatibility Complex Class II Region
Source: Front Genet. 2021 May 28;12:665899. doi: 10.3389/fgene.2021.665899 (PMC8193847; doi:10.3389/fgene.2021.665899)

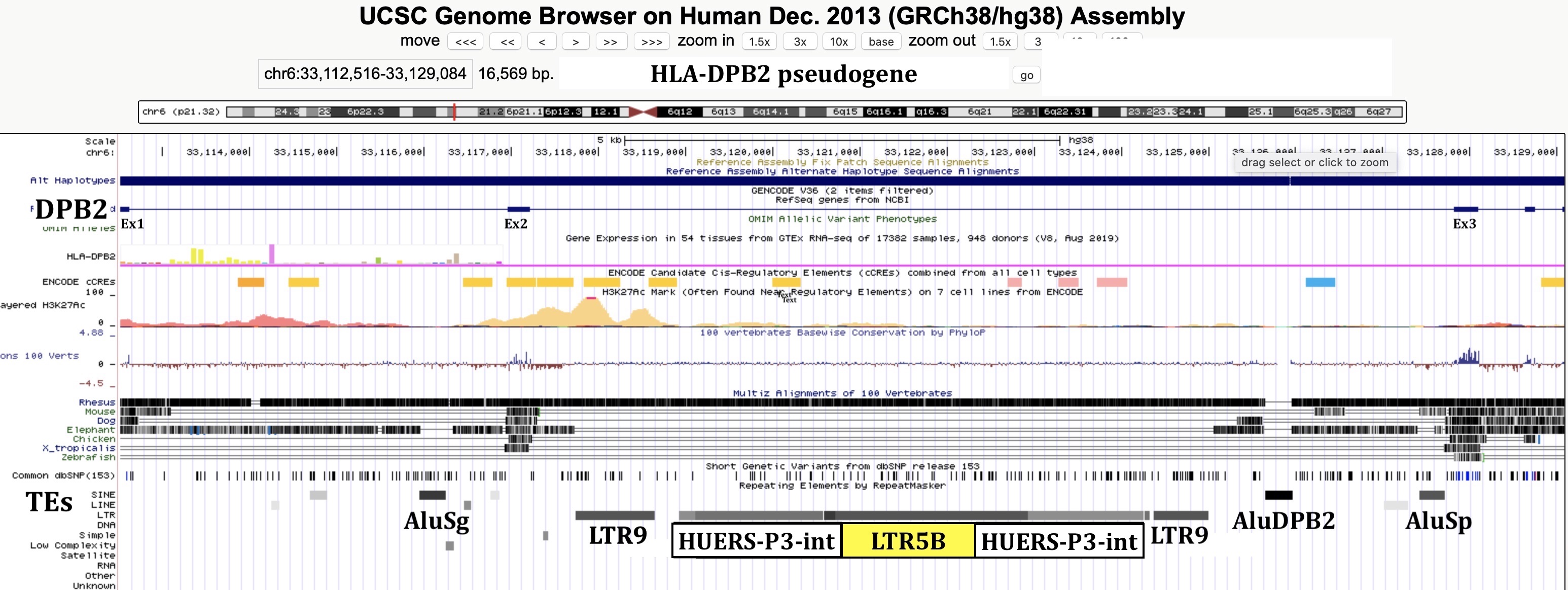

Supplement: Supplementary file 11 [file Image_1.JPEG]

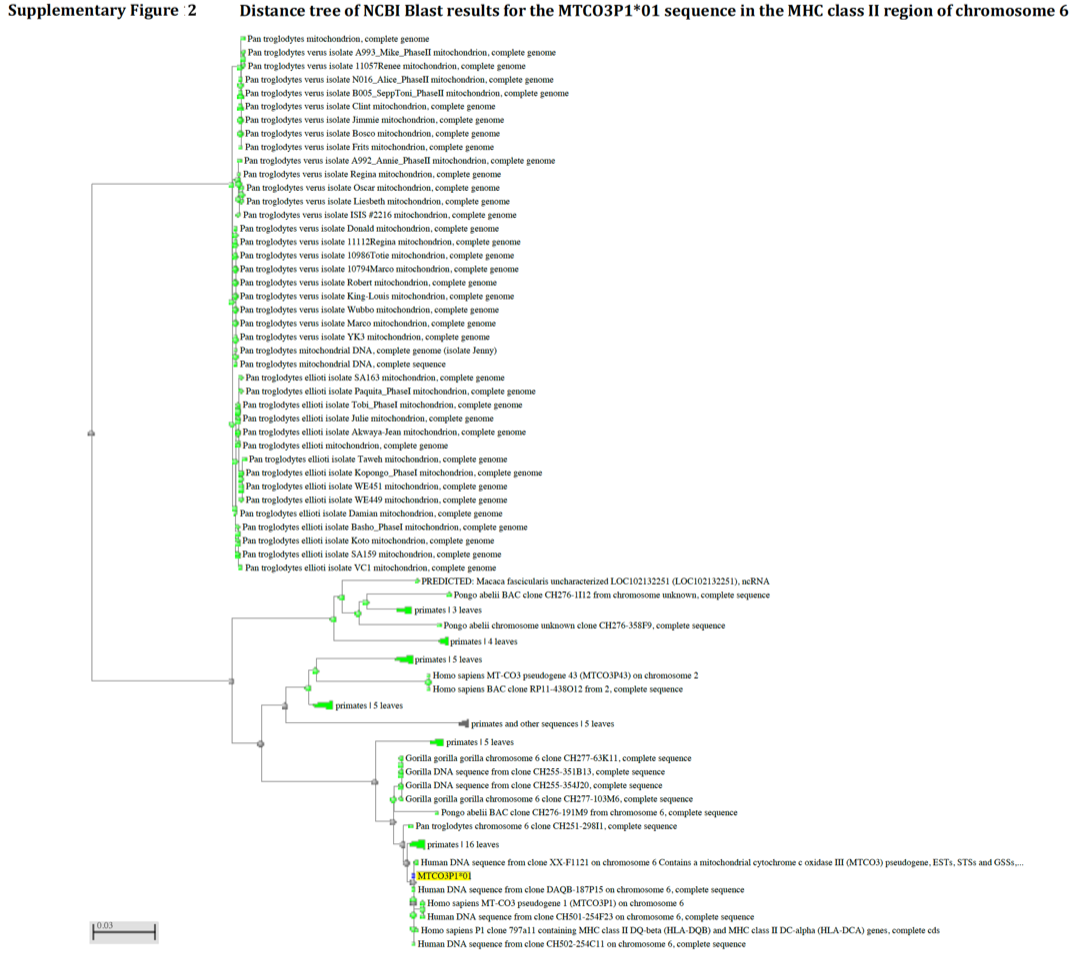

Supplement: Supplementary file 12 [file Image_2.TIFF]
